# Extensive Unexplored Human Microbiome Diversity Revealed by Over 150,000 Genomes from Metagenomes Spanning Age, Geography, and Lifestyle

**DOI:** 10.1016/j.cell.2019.01.001

**Published:** 2019-01-24

**Authors:** Edoardo Pasolli, Francesco Asnicar, Serena Manara, Moreno Zolfo, Nicolai Karcher, Federica Armanini, Francesco Beghini, Paolo Manghi, Adrian Tett, Paolo Ghensi, Maria Carmen Collado, Benjamin L. Rice, Casey DuLong, Xochitl C. Morgan, Christopher D. Golden, Christopher Quince, Curtis Huttenhower, Nicola Segata

**Affiliations:** 1 CIBIO Department, University of Trento, Trento, Italy; 2 Institute of Agrochemistry and Food Technology-National Research Council, Valencia, Spain; 3 Harvard University, Cambridge, MA, USA; 4 Harvard T.H. Chan School of Public Health, Boston, MA, USA; 5 University of Otago, Otago, New Zealand; 6 Warwick Medical School, University of Warwick, Warwick, UK; 7 The Broad Institute, Cambridge, MA, USA

**Keywords:** human microbiome, metagenomics, metagenomic meta-analysis, metagenomic assembly, non-Westernized microbiomes, unexplored microbial diversity, metagenomic mappability

## Abstract

The body-wide human microbiome plays a role in health, but its full diversity remains uncharacterized, particularly outside of the gut and in international populations. We leveraged 9,428 metagenomes to reconstruct 154,723 microbial genomes (45% of high quality) spanning body sites, ages, countries, and lifestyles. We recapitulated 4,930 species-level genome bins (SGBs), 77% without genomes in public repositories (unknown SGBs [uSGBs]). uSGBs are prevalent (in 93% of well-assembled samples), expand underrepresented phyla, and are enriched in non-Westernized populations (40% of the total SGBs). We annotated 2.85 M genes in SGBs, many associated with conditions including infant development (94,000) or Westernization (106,000). SGBs and uSGBs permit deeper microbiome analyses and increase the average mappability of metagenomic reads from 67.76% to 87.51% in the gut (median 94.26%) and 65.14% to 82.34% in the mouth. We thus identify thousands of microbial genomes from yet-to-be-named species, expand the pangenomes of human-associated microbes, and allow better exploitation of metagenomic technologies.

## Highlights

• Large-scale metagenomic assembly uncovered thousands of new human microbiome species
• The new genome resource increases the mappability of gut metagenomes over 87%
• Some of the newly discovered species comprise thousands of reconstructed genomes
• Non-Westernized populations harbor a large fraction of the newly discovered species

## Graphical Abstract

**Figure:**
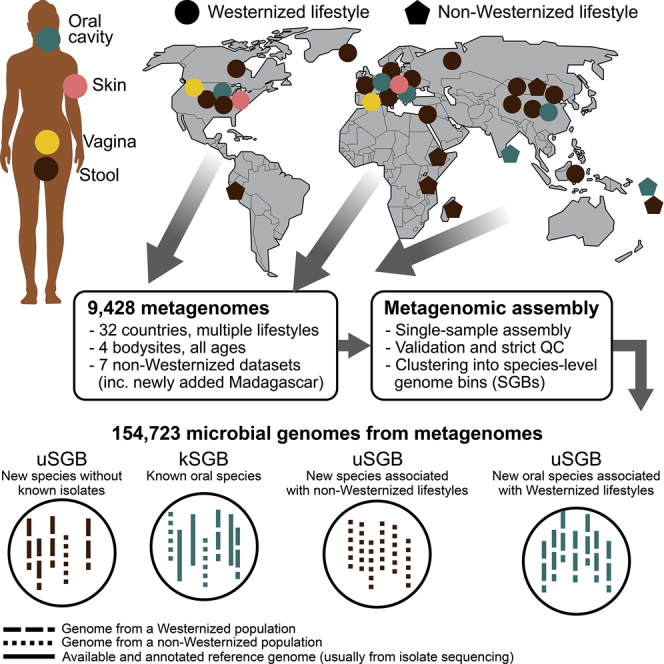

## Teaser

The human microbiome harbors many unidentified species. By large-scale metagenomic assembly of samples from diverse populations, we uncovered >150,000 microbial genomes that are recapitulated in 4,930 species. Many species (77%) were never described before, increase the mappability of metagenomes, and expand our understanding of global body-wide human microbiomes.

## Introduction

Despite extensive recent studies of the human microbiome using a variety of culture-independent molecular technologies (Human Microbiome Project Consortium, 2012, Qin et al., 2010, Quince et al., 2017a, Rinke et al., 2013), most characterization of these ecosystems is still focused on microbes that are easily cultivable, particularly when those with sequenced isolate genomes are considered. Since physiological characterization of diverse, uncharacterized human-associated microbes by cultivation can be difficult in high throughput (Browne et al., 2016), additional approaches are needed that scale with the extent of populations that can now be surveyed using metagenomic sequencing. Culture-independent genomic approaches that are scalable to large cohorts (Human Microbiome Project Consortium, 2012, Qin et al., 2010, Quince et al., 2017a) have facilitated access to an expanded set of isolation-recalcitrant members of the microbiome, but they also suggested the presence of a large fraction of still unexplored diversity (Nielsen et al., 2014, Rinke et al., 2013).

Here, we present a set of 154,723 microbial genomes that are often prevalent, population specific, and/or geographically specific that we reconstructed via single-sample assembly from a total of 9,428 global, body-wide metagenomes. Other studies have also succeeded in reconstructing microbial genomes by metagenomic assembly on single human cohorts (Bäckhed et al., 2015, Brooks et al., 2017, Ferretti et al., 2018, Human Microbiome Project Consortium, 2012, Raveh-Sadka et al., 2015, Sharon et al., 2013), but systematic cross-study cataloging of metagenomically assembled genomes focused so far on non-human environments (Oyama et al., 2017, Parks et al., 2017). Complementary techniques, such as co-abundance of gene groups (Nielsen et al., 2014), can identify genomic bins without reference, but these techniques do not account for sample-specific strains and strain-level differences in the sequence reconstruction and thus require downstream single-nucleotide variation analysis on specific genomic regions to uncover strain variability (Quince et al., 2017b, Truong et al., 2017).

Using large-scale single-sample metagenomic assembly supported by strict quality control (including filtering based on nucleotide polymorphisms), we identified 3,796 species-level clades (comprising 34,205 genomes) without previous whole-genome information. This identified several taxa prevalent but previously unobserved even in well-profiled populations (e.g., a genus-level Ruminococcaceae clade phylogenetically close to *Faecalibacterium*), extensive taxonomically uncharacterized species associated with non-Western populations, and the presence of several taxa from undersampled phyla (e.g., Saccharibacteria and Elusimicrobia) in oral and gut microbiomes. The resulting genome set can thus serve as the basis for future strain-specific comparative genomics to associate variants in the human microbiome with environmental exposures and health outcomes across the globe.

## Results

### Recovering Over 150,000 Microbial Genomes from ∼10,000 Human Metagenomes

**Figure S1:**
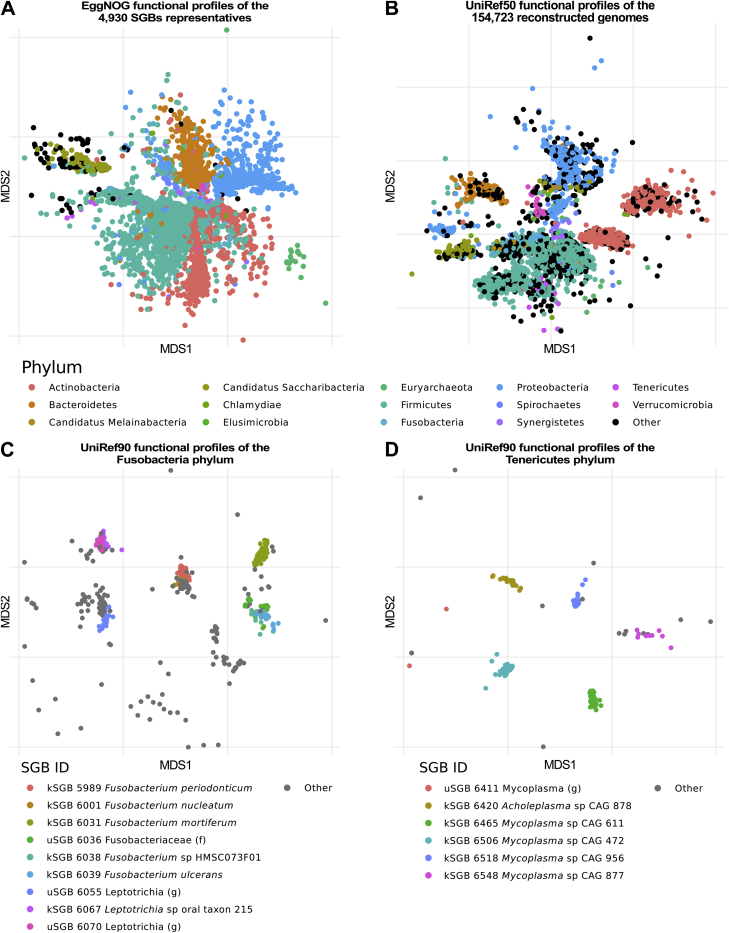
Overview of the Functional and Metabolic Annotations of the Representatives of the SGBs and of the Whole Set of 154,723 Reconstructed Genomes, Related to Figure 1 (A) Ordination plot of the KEGG gene families annotated using eggNOG (see STAR Methods) of the 4,930 SGBs’ representatives, colored by the 14 most represented phyla. (B) Ordination plot of the UniRef50 gene families present in the 154,723 reconstructed genomes as annotated by mapping the genomes against both Uniref90 and Uniref50 (see STAR Methods). Ordination plots of the UniRef90 gene families for all the reconstructed genomes assigned to the (C) Fusobacteria and (D) Tenericutes phyla are also reported as examples of fine-grained functional differentiation.

We employed a very large-scale metagenomic assembly approach to reconstruct bacterial and archaeal genomes populating the human microbiome (see STAR Methods). From a total of 9,316 metagenomes spanning 46 datasets from multiple populations, body sites, and host ages (Table S1), and an additional cohort from Madagascar (Golden et al., 2017) (STAR Methods; Table S1), we reconstructed a total of 154,723 genomes (each made up of a group of clustered contigs; see STAR Methods) using a single-sample assembly strategy tailored at maximizing the quality rather than the quantity of genomes reconstructed from each sample. The resulting catalog greatly expands the set of ∼150,000 microbial genomes publicly available (see STAR Methods). All assembled genomes passed strict quality control including estimation of completeness, contamination, and a measure of strain heterogeneity (see STAR Methods), and they exceed the thresholds to be defined medium quality (MQ) according to recent guidelines (Bowers et al., 2017) (completeness >50%, contamination <5%). The quality of these genomes was comparable with that of isolate sequencing (STAR Methods; Table S2) and in line also with the quality achievable by manually curated metagenomic approaches (Table S2) and time-series or cross-sectional metagenomic co-binning (see STAR Methods; Table S2). Genomes may include contigs from plasmids (see STAR Methods), and stricter quality control reduced the set of near-complete, high-quality (HQ) genomes to 70,178 with completeness higher than 90% and reduced probability of intra-sample strain heterogeneity (<0.5% polymorphic positions, see STAR Methods). The main characteristics of HQ genomes are in line and in some cases better than those from the compendium of reference genomes available in public repositories, although MQ genomes also had similar quality scores compared to HQ genomes (modulo completeness; STAR Methods). The set of genomes we reconstructed (Table S3; Data and Software Availability) and the associated 2.85 million (M) total functional annotations (STAR Methods; Figure S1) are thus appropriate as a basis for more in-depth microbial community analyses.

### Human Microbiome Genomes Belong to ∼5,000 Functionally Annotated SGBs

**Figure 1:**
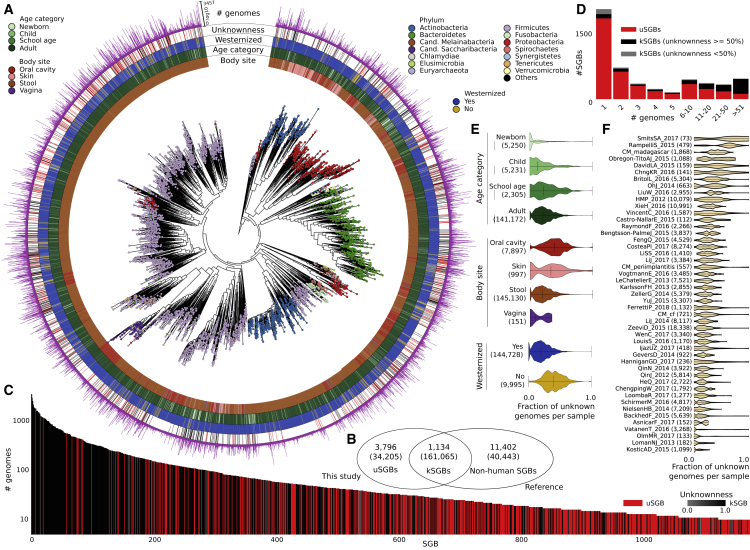
4,930 SGBs Assembled from 9,428 Meta-analyzed Body-wide Metagenomes (A) A human-associated microbial phylogeny of representative genomes from each species-level genome bin (SGB). Figure S3A reports the same phylogeny but including isolate genomes not found in the human-associated metagenomes. (B) Overlap of SGBs containing both existing microbial genomes (including other metagenomic assemblies) and genomes reconstructed here (kSGBs), SGBs with only genomes reconstructed here and without existing isolate or metagenomically assembled genomes (uSGBs), and SGBs with only existing genomes and no genomes from our metagenomic assembly of human microbiomes (non-human SGBs). (C) Many SGBs contain no genomes from sequenced isolates or publicly available metagenomic assemblies (uSGBs). Only SGBs containing >10 genomes are shown. (D) Fraction of uSGBs and kSGBs as a function of the size of the SGBs (i.e., number of genomes in the SGB). (E) Distribution of the fraction of uSGBs in each sample by age category, body site, and lifestyle. (F) Distribution of the fraction of uSGBs in each study.

**Figure S2:**
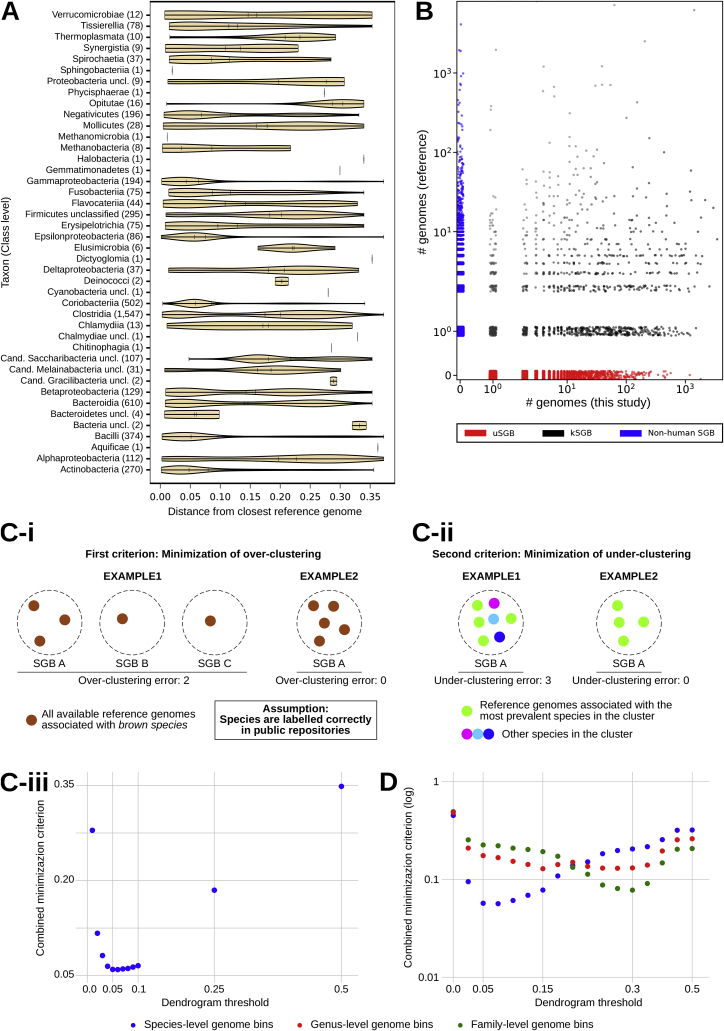
Overview of the Reconstructed SGBs and Criteria for SGB Definition and Taxonomic Assignment, Related to Figure 6 (A) Distribution of the distances of each reconstructed genome to the closest available isolate genomes, grouped by the class assigned to the matching isolate genomes. (B) The 4,930 identified species-level genome bins (SGBs) comprise a very variable fraction of already available genomes versus genomes we reconstructed from metagenomes. (C) Minimization criterion adopted to find the optimal cutoff in the hierarchical clustering of genomes to define SGBs. Two criteria are taken into account: minimization of the over-clustering error (C-i), and minimization of the under-clustering error (C-ii). Results showed a minimization of the error for a threshold equal to 0.05 (C-iii), which was thus adopted to discretize subtrees in the dendrogram and generate SGBs spanning ∼5% genetic diversity. (D) The same minimization criterion reported in (C-iii) for species-level bins is also adopted to identify the genomic diversity for genus-level and family-level bins.

To organize the 154,723 genomes into species-level genome bins (SGBs), we employed an all-versus-all genetic distance quantification followed by clustering and identification of genome bins spanning a 5% genetic diversity, which is consistent with the definition of known species (see STAR Methods) and with other reports (Jain et al., 2018). We obtained 4,930 SGBs from 22 known phyla (Figure 1A; Table S4). This is likely an underestimate of the total phylum-level diversity, because some SGBs are very divergent from all previously available reference genomes and cannot be confidently assigned to a taxonomic family (Table S4): 345 SGBs (58% of which with HQ or multiple reconstructed genomes) display more than 30% Mash-estimated genetic distance (Ondov et al., 2016) from the closest isolate with a phylum assignment (Figure S2A). The SGB genomic catalog spans on average 3.0%, SD 1.8% intra-SGB nucleotide genetic variability, and each SGB contains up to 3,457 genomes from different individuals (average 31.4, SD 147.6; Figures 1C and S2B).

Functional annotation of all the reconstructed genomes assigned a UniRef90 (The UniProt Consortium, 2017) label to 230 M genes and a UniRef50 to 268 M genes (72.7% and 84.8% of the total of 316 M genes, respectively). Additional EggNOG (Huerta-Cepas et al., 2017) labels were assigned to 80.8% of the 4,930 SGBs’ genome representatives. The functional potential profiles of the genomes had, as expected, clear phylogenetic differentiation (Figure S1), and the rate of annotation varied greatly in SGBs (e.g., >90% genes annotated for well-studies species such as *Escherichia coli* or *Bacteroides fragilis* versus 22% for ID 15286, which is the largest SGB without reference genomes). Each of the body sites considered had a clear distinctive set of annotations with the adult fecal microbiome enriched for 101,056 gene families (Table S5, Bonferroni-corrected Fisher's test p < 0.01), representative of anaerobe-specific functions such as formate oxidation and methanogenesis and a strong representation of biofilm formation functions in the oral cavity and on the skin. Genomes from the stool microbiome of newborns had 94,562 enriched gene families (Table S5, Bonferroni-corrected Fisher's test p < 0.01) comprising a variety of functions such as folate biosynthesis and lactose, oligosaccharides, and mucin degradation that are typical of the niche and nutritional regime of unweaned infants (Asnicar et al., 2017, Marcobal et al., 2011, Yatsunenko et al., 2012). Age-specific functions (Table S5) are characterized by the later host developmental stages of children (17,121 specific functions) and school-age individuals (349 specific functions). The Westernization process has also a strong influence on the functions encoded in the stool microbiome, with a total of 106,872 differential families (Table S5, Bonferroni-corrected Fisher's test p < 0.01) spanning enzymes involved in the metabolism of complex carbohydrates, such as xylose and cellulose, and in specific cobalamin biosynthesis pathways; these are likely reflecting dietary habits, among other environmental differences. The organization of the reconstructed genomes in SGBs and their functional profiling will be the basis for comprehensive future metagenomic characterizations.

### The Reconstructed Genomes and SGBs Increase the Diversity and Mappability of the Human Microbiome

**Figure S3:**
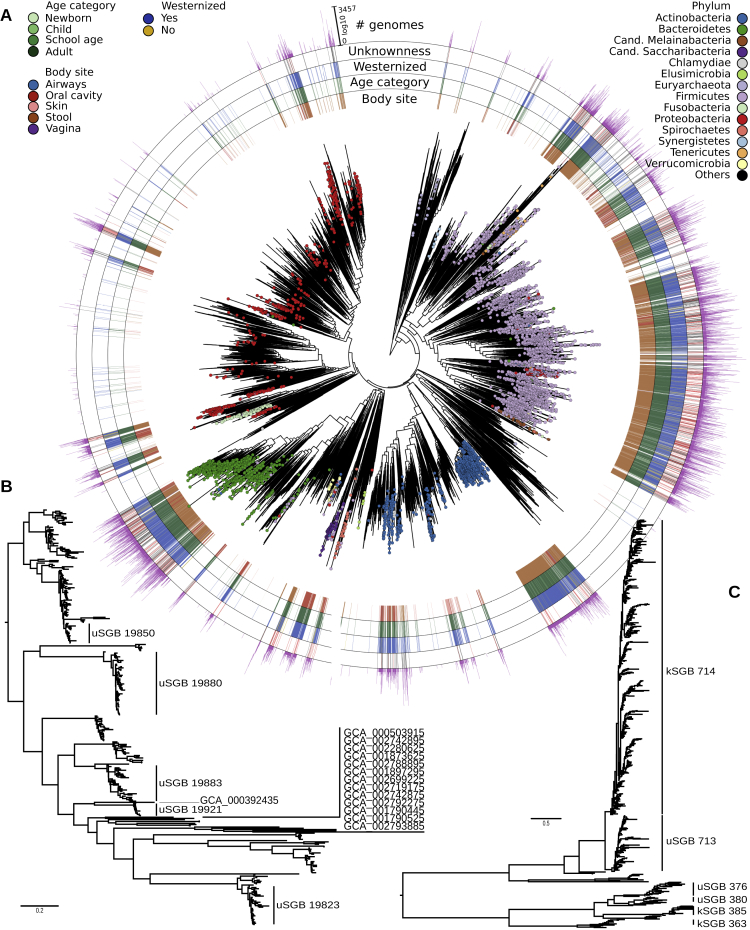
Phylogenetic Trees for All SGBs and Reference Genomes and Subtrees of Saccharibacteria and Archaea, Related to Figure 1 (A) Phylogenetic tree that includes the representatives of the SGBs presented in Figure 1A together with all the non-human bins (represented in white in the external rings), for a total of 16,332 genomes (15,299 after the internal quality control in PhyloPhlAn). (B) Phylogenetic tree of the 337 reconstructed genomes taxonomically assigned to the candidate phylum Saccharibacteria present in the 108 SGBs, including available reference genomes (publicly available reference genomes are labeled with the “GCA” prefix). (C) Phylogenetic tree of the 675 archaeal genomes reconstructed in this study. 487 genomes belong to the *Methanobrevibacter smithii* kSGB (ID 714).

We identified 3,796 SGBs (i.e., 77.0% of the total) covering unexplored microbial diversity as they represent species without any publicly available genomes from isolate sequencing or previous metagenomic assemblies (Figures 1B and S3A). These SGBs, that we named unknown SGBs (uSGBs), include on average 9.0, SD 45.4 reconstructed genomes, and 1,693 of them (45%) had at least one HQ genome. Recursive clustering of SGBs’ representatives at genus- and family-level genetic divergence (see STAR Methods) provided taxonomic context for 75.2% of the uSGBs with 1,472 assignments to genera and 1,383 more to families (Table S4). The 941 uSGBs that were left unplaced at family level remained unassigned for limitations of whole-genome similarity estimates, but we report the similarity and taxonomy of the closest matching strain (Table S4).

Only 1,134 of the 4,930 SGBs represent at least partially known SGBs (kSGBs) that include one or more genomes in public databases. This number of kSGBs is consistent with the 1,266 species we found at least once in the same set of metagenomes (Pasolli et al., 2017) at >0.01% abundance using reference-based taxonomic profiling (Truong et al., 2015). Most uSGBs represent instead relatively rare human-associated microbes (46.7% of uSGBs comprise one reconstructed genome only, Table S4, and 46.1% genomes in uSGBs are at <0.5% relative abundance, STAR Methods and Table S4), but some uSGBs are highly prevalent, with 10 uSGBs in the set of the 100 SGBs with the largest number of reconstructed genomes (Figures 1C, 1D, and S2B) and 368 genomes in uSGBs accounting for >10% of reads. Because many uSGBs are associated with specific sample types (e.g., oral cavity or non-Westernized samples, Figure 1E), the actual number of possibly redundant genomes they contain is likely underestimated for those sample types with comparably fewer metagenomes available. Functional annotation of uSGB genomes assigned a UniRef90 cluster to only 31.9% of the genes, while the annotation rate increased to 81.0% for kSGB genomes.

**Figure 2:**
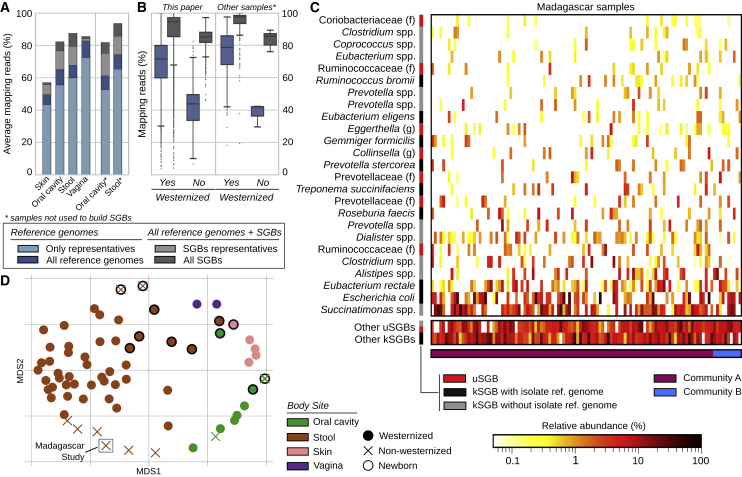
The Expanded Genome Set Substantially Increases the Mappability of Human Metagenomes (A) We mapped the subsampled original 9,428 metagenomes and 389 additional samples not considered for building the SGBs against the 154,723 reconstructed genomes and 80,990 previously available genomes. Raw-read mappability increased significantly (Mann-Whitney U test, p < 1e−50), e.g., from an average of 67.76% to 87.51% in the gut. Representative genomes refer to the highest-quality genomes selected from the 4,930 human SGBs and the 11,402 non-human SGBs. Extended statistics are in Figure S4. (B) Metagenomic read mappability increases more in non-Westernized than Westernized gut microbiomes (Welch's t test, p < 1e−50), both when considering samples used for SGBs’ reconstruction (26.50% average increase in 7,059 Westernized samples versus 96.56% in 454 non-Westernized samples) and when considering 264 additional samples not used for SGBs’ reconstruction (25.16% versus 117.40% average increase, respectively). (C) The gut microbiomes from Madagascar we sequenced here showed several highly abundant uSGBs and a large set of SGBs reconstructed in only subsets of the samples. Many kSGBs in this dataset do not contain isolate genomes but only previous metagenomic assemblies. The 25 most abundant SGBs are reported and ordered according to their average relative abundance. (D) Multidimensional scaling on datasets using the Bray-Curtis distance on per-dataset SGB prevalences highlights distinct microbial communities between Westernized and non-Westernized populations within and between body sites and age categories.

**Figure S4:**
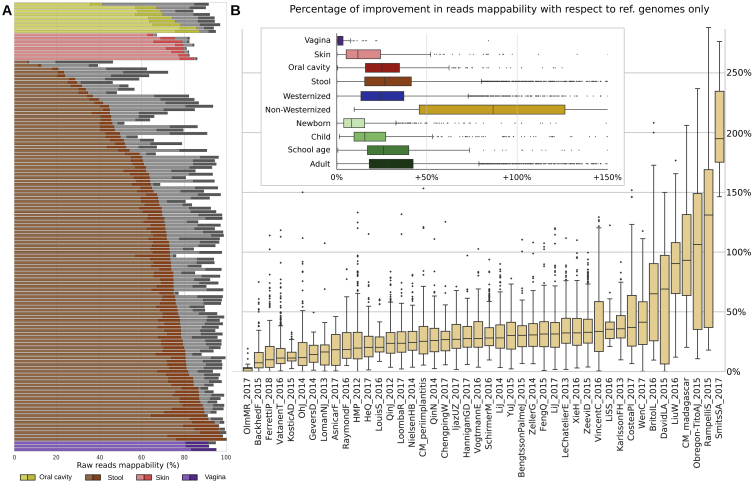
Improvement of Read Mappability Statistics by Considering the Set of Microbial Genomes We Assembled in This Work, Related to Figure 2 (A) Fraction of reads that can be mapped against different sets of genomes from isolate sequencing and the metagenomically reconstructed genomes. A subset of 132 full (i.e., not subsampled) metagenomes is shown (3 metagenomes randomly selected from each study). Samples are colored and grouped by body site. The colored part of the bar refers to the reads that can be mapped against a previously available reference genome, while the gray bars extend to highlight the total mappability we achieved using the 154,723 microbial genomes reconstructed in this study. (B) Percentage of increase in the mappability when using also the 154,723 reconstructed SGBs to map metagenomic reads. Boxplots represent values grouped by body site, lifestyle, age category (upper panel) and study (lower panel). The percentage of improvement is calculated with respect to the fraction of reads that could map using only and all the reference genomes. All the 9,428 metagenomes used in this study were mapped after being subsampled at 1% (see STAR Methods). Averaged statistics are reported in Figures 2A–2B.

The expanded human microbiome diversity induced by the uSGBs (200% increase in the reconstructed phylogenetic branch length, 50% considering only uSGBs with >10 genomes, Figure 1A) can be crucial as a genomic reference in the characterization (“mappability”) of the sequence information in a metagenome. Genomes in uSGBs are indeed responsible for a substantial decrease of the metagenomic reads that do not match any microbial reference (Figures 2A and S4). This is due both to uSGBs representing target microbes without assigned species (16.76% average increase using only representative genomes of uSGBs, Figure 2A) and to the expansion of pangenomes of kSGBs and uSGBs (27.84% increase when considering all genomes instead of only SGB representatives). On average, the read mappability for stool samples reached 87.51% (29.14% increase, Figure 2A) and 82.34% in the oral cavity (26.40% increase, Figure 2A). Some outlier samples decreased the averages as the median final mappabilities were higher, reaching 94.26% for the stool microbiome and 90.13% for the oral microbiome in Westernized populations. The mappability of the skin microbiome was also increased (15.17% increase) but reached a lower overall value (57.07%) because fewer skin samples were available and non-bacterial organisms such as the molluscum contagiosum virus (Oh et al., 2014) and fungi from the *Malassezia* genus (Tett et al., 2017) also populate the skin. Mappability in the vaginal microbiomes was instead already high (82.77%) due to a reduced panel of known species dominating the large majority of these communities, but the set of 4,930 reconstructed SGBs still increased the mappability by 3.42%. The mappability increase is dramatic for the gut microbiomes of non-Westernized populations that are very poorly represented by available reference genomes (42.33% mappability) and can now reach a mappability of 83.20%, which is comparable with that of Westernized populations (Figure 2B). These substantial gains in read mappability when using our genome catalog are achieved also for stool and oral samples not used to construct the resource (STAR Methods; Figures 2A and 2B), confirming its relevance as reference for future studies.

SGBs without publicly available genomes (uSGBs) represent 34,205 reconstructed genomes (Figure 1B), belonging to metagenomes in different body sites, ages, and general lifestyles (Figures 1E and 1F). Microbiomes with lower diversity, such as those from infants or the female urogenital tract, carried a generally lower fraction of uSGBs. Populations with non-Westernized lifestyles—including the Madagascar cohort we sequenced (Figures 2C and 2D)—conversely yielded a fraction of genomes in uSGBs nearly double that of Western-style populations (average 40% and 21%, respectively, p < 1e−50, Figure 1E). Most of the abundant kSGBs in the Madagascar cohort do not include isolate genomes but only sequences from previous metagenomic assemblies (Figure 2C), and these uSGBs and poorly characterized kSGBs are contributing to the clear distinction of the gut microbiome with respect to general lifestyles (Figure 2D). The higher rate of uSGB recovery in non-Westernized populations is likely the consequence of comparatively fewer studies profiling these populations and their more diverse gut microbiomes.

### The Diversity of Human-Associated Archaea and Bacterial Phyla Is Expanded by uSGBs

Many clades, including some phyla, were greatly expanded by reconstructed genomes belonging to species that do not have deposited genome sequences or taxonomic labels (uSGBs). For example, the candidate phylum Saccharibacteria (previously named TM7) contains members of the oral microbiome that are particularly difficult to cultivate (He et al., 2015, Solden et al., 2016). For this clade, we reconstructed 387 genomes from 108 SGBs (Figure 1A), some representing members observed only using 16S rRNA gene sequencing (Brinig et al., 2003, Segata et al., 2012a). An isolate reference genome was only available for a single SGB within this clade (ID 19849); the other 16 reference genomes for this phylum were undetected in oral cavity metagenomes (Figure S3B). The 107 Saccharibacteria uSGBs thus suggest a substantially undersampled diversity of human-associated members of this phylum. Its importance is also confirmed by the occurrence of at least one genome from these 108 SGBs in 33% of oral cavity samples, where they can reach average abundances above 3% (Table S4) and maximum abundances exceeding 10%.

We further recovered 675 genomes of Archaea (526 from 6 kSGBs and 149 from 13 uSGBs, Figure 1A) and reconstructed its phylogeny (Figure S3C). More than half of these genomes (n = 487) belonged to the *Methanobrevibacter smithii* kSGB (ID 714), which was present at relatively low abundance (average 1.06%, SD 1.26%). A related but diverged SGB including 94 genomes was identified (ID 713, 5.6% nucleotide divergence from the *M. smithii* isolate genome) at comparable abundance (average 0.92%, SD 2.02%), but it notably accounted for up to 20% of all reads in some gut samples. Among uSGBs, we also reconstructed genomes assigned to *Thermoplasmatales* (ID 376, 378, 380, 381), Candidatus *Methanomethylophilus* (ID 372, 382, 384), *Methanomassiliicoccus* (ID 362, 364), and *Methanosphaera* (ID 697), all very distant from their nearest reference genomes (average 22.4%, SD 4.0% nucleotide distance). This expanded human-associated archaeal diversity suggests the presence of several as-yet-uncharacterized archaea of potentially unique functional relevance in this ecosystem.

### Several Prevalent Uncharacterized Intestinal Clostridiales Clades Occur Phylogenetically between Ruminococcus and Faecalibacterium

**Figure 3:**
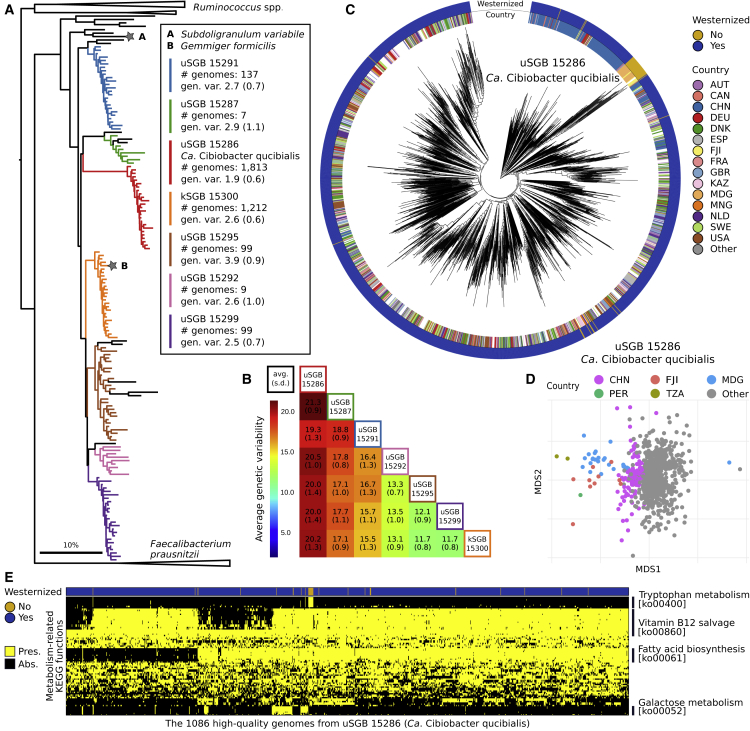
Several Prevalent Intestinal uSGBs Are Found within the Clostridiales Order Related to *Ruminococcus* and *Faecalibacterium* (A) All SGBs in the assembled phylogeny (Figure 1A) placed between reference genomes for *Ruminococcus* and *Faecalibacterium* species that are reported as collapsed trees. A maximum of 25 HQ genomes from each SGB are displayed, and SGBs with <3 genomes are left black. (B) The monophyletic clade with the six uSGBs and the kSGB containing *Gemmiger formicilis* represent clearly divergent species with inter-species genetic distance typical of genus-level divergence (average 16.6%, SD 3.1% nucleotide distance). (C) A whole-genome phylogeny for the 1,806 genomes in *Ca.* Cibiobacter qucibialis (STAR Methods). Some subtrees associate with geography and non-Westernized populations, while others seems to be geography- and lifestyle-independent (see text). (D) Multidimensional scaling of genetic distances among genomes of *Ca.* Cibiobacter qucibialis highlights the divergence of strains carried by non-Westernized populations, with Chinese populations subclustering within the large cluster of Westernized populations. (E) Madagascar-associated strains of *Ca.* Cibiobacter qucibialis (uSGB 15286) uniquely possess the *trp* operon for tryptophan metabolism (Table S7). Other functional clusters in Westernized strains from geographically heterogeneous populations include vitamin B12 and fatty acid biosynthesis and galactose metabolism. The KEGG functions present in >80% or in <20% of the samples were discarded except for significant associations with lifestyle.

**Figure S5:**
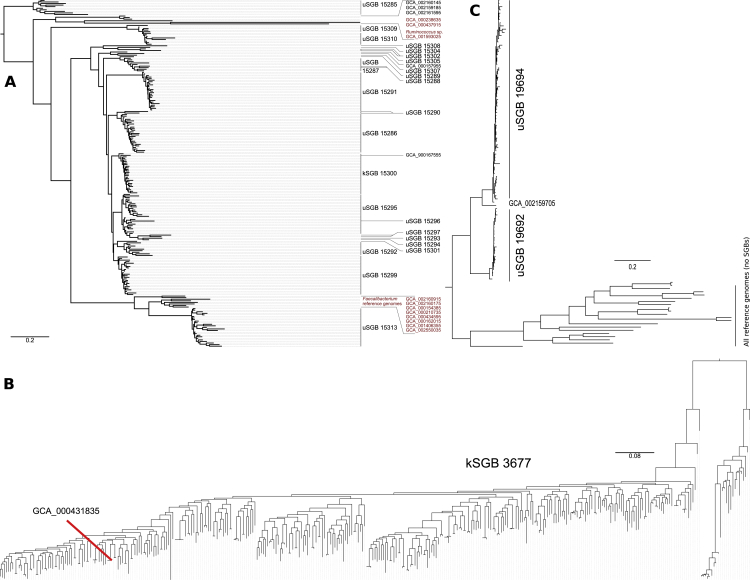
Phylogenetic Trees for SGBs Placed between *Ruminococcus* and *Faecalibacterium*, Succinatimonas kSGB (ID 3677), and Two Elusimicrobia uSGBs, Related to Figure 3 and 5 (A) Phylogenetic tree of SGBs placed between reference genomes for *Ruminococcus* and *Faecalibacterium* species in Figure 1A (highlighted in red), as already reported in Figure 3A but without collapsed branches and including the two reference genomes GCA_000238635 and GCA_000437915 (also highlighted), originally labeled as *Subdoligranulum* sp. 4_3_54A2FAA and *Subdoligranulum* sp. CAG:314, respectively. (B) Phylogenetic tree of the Succinatimonas kSGB (ID 3677) including the only available reference genome. (C) Phylogenetic tree of the two Elusimicrobia uSGBs enriched in non-Westernized populations and of all the available Elusimicrobia reference genomes.

Some of the uSGBs with the largest number of reconstructed genomes are also highly abundant in the gut microbiome, with 1,153 uSGBs totaling >13,000 genomes each present in the sample where it has been reconstructed at an average abundance >1% (and 172 uSGBs at >5% average abundance). Among them, uSGB ID 15286, that we named “*Candidatus* Cibiobacter qucibialis”, is the most prevalent uSGB, comprising 1,813 reconstructed genomes. This species is phylogenetically placed between *Faecalibacterium* and *Ruminococcus* (Figures 3A and S5A), key members of the gut microbiome that are typically present at comparably lower abundances (1.84% *Faecalibacterium* kSGB and 1.29% *Ruminococcus* kSBG in contrast to 2.47% *Ca.* Cibiobacter qucibialis). Six other prevalent (1,563 total genomes) and abundant (1.14% average abundance) SGBs occurred monophyletically in the same subtree between faecalibacteria and ruminococci (Figure 3A). Only one of these seven total SGBs contains an isolate genome, which is the recently sequenced *Gemmiger formicilis* genome (Gossling and Moore, 1975) included in kSGB ID 15300 (1,212 genomes, Figures 3A and 3B). A genome from the *Subdoligranulum variabile* species, itself not found in any of the study’s assemblies, was the only other reference phylogenetically close to this clade, explaining the previous identification of an unknown *Subdoligranulum* (“*Subdoligranulum* unclassified”) as the most prevalent single taxon in reference-based profiles of the gut microbiome (Pasolli et al., 2017). This prevalent 7-SGBs clade comprising 3,370 reconstructed genomes that can be very abundant (>5% relative abundance in >200 samples) is thus an important but so far neglected genus-level lineage in the human microbiome.

In an estimated maximum-likelihood whole-genome phylogeny of the 1,813 genomes belonging to *Ca.* Cibiobacter qucibialis (Figure 3C), genomes of non-Westernized populations were placed together in a monophyletic subtree (Figure 3C). This subtree included 26 strains from the Madagascar microbiomes we sequenced in this work, in addition to strains from three other populations with traditional lifestyles but differing geographic locations (Figure 3D). Although the non-Westernized subtree includes few genomes (2% of the total), this is a consequence of limited sampling from these population types because the prevalence of this SGB in Westernized populations is comparable (23% against 15% in non-Westernized populations). No clear internal clustering was evident for Westernized samples (Figure 3C), except for a large set of 222 samples retrieved from the seven Chinese cohorts that are monophyletically placed in the same subtree despite widely different pre-sequencing protocols (Table S6) and resemble non-Westernized genomes (Figures 3C and 3D). This suggests a complex process of gut microbial ecological establishment in which both host lifestyle and biogeography play roles with comparable effect sizes.

Functional potential profiling of SGBs can suggest metabolic features that distinguish each clade, and for *Ca.* Cibiobacter qucibialis, we found functional modules specific to only some of the constituent strains (Figure 3E; Table S7). These include the pathway for the biosynthesis of vitamin B12 from precorrin-2, lacking in some Westernized strains that instead use other pathways for vitamin B12 production, as well as gene clusters devoted to fatty acid biosynthesis and galactose metabolism (Figure 3E). A strong lifestyle-associated difference characterized the non-Westernized strains in *Ca.* Cibiobacter qucibialis (uSGB 15286), as they were the only strains in this SGB with the whole set of genes in the *trp* operon for tryptophan metabolism. The Trp biosynthetic pathway can be organized as whole-pathway operon or as dispersed genes in different bacterial species (Merino et al., 2008), as a result of organismal divergence, adjustment to environmental availability of key molecules, and lateral gene transfer events (Xie et al., 2003). We speculate that the presence of the whole operon in the non-Westernized strains may be indicative of divergent evolution in the Westernized strains of *Ca.* Cibiobacter qucibialis, potentially as a consequence of a loss-of-operon event.

### Sample-Specific Strain Recovery Greatly Enlarges the Pangenomes of Key Intestinal Microbes

**Figure 4:**
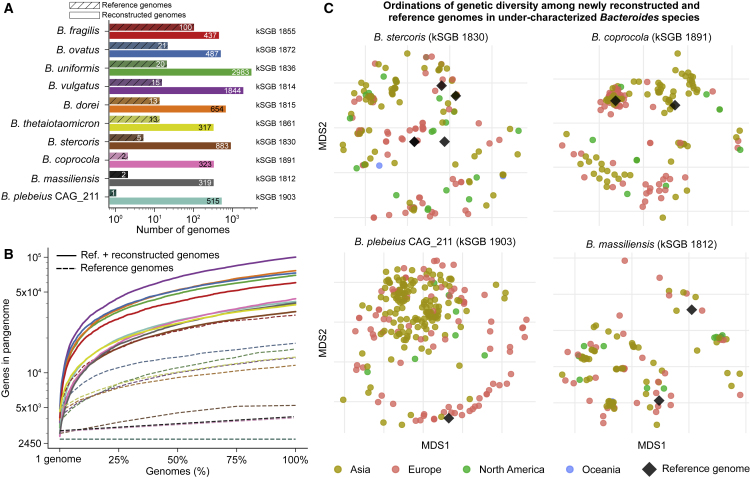
The Metagenomically Reconstructed Genomes Greatly Expand the Genetic and Functional Diversity of the Ten *Bacteroides* Species Most Prevalent in the Human Gut (A) Additional *Bacteroides* genomes we assembled from metagenomes increase the size of the ten most prevalent *Bacteroides* kSGBs from 4 to >500 times. (B) The expanded *Bacteroides* kSGBs account for much larger pangenomes that capture a greater functional potential. (C) Ordinations on intra-SGB genetic distances (fractions of nucleotide mutations in the core genome) highlight the genetic structure of *Bacteroides* species and that reference genomes were available only for a reduced subset of subspecies structures (additional ordinations are in Figure S6A).

*Bacteroides* are among the most studied intestinal species (Marcobal et al., 2011) and are core in European and American populations (Human Microbiome Project Consortium, 2012, Nielsen et al., 2014), but our analysis still recovered unsampled intra-species diversity. Among the ten largest SGBs, the number of available isolate genomes ranges from 1 (*Bacteroides plebeius*) to 100 (*Bacteroides fragilis*), whereas we added from 317 to 2,983 individual representatives (Figure 4A). These expanded genome sets provide much larger collections of distinct genes that can be present in strains of each species, i.e., pangenomes, which spanned ∼30,000 to >70,000 genes per *Bacteroides* species, capturing a substantially wider functional potential compared to isolate genomes (Figure 4B). The number of genomes in a species bin did not correlate well with the size of the associated pangenome (Pearson correlation 0.48, p = 0.16), indicating that pangenome recovery is not simply a function of the amount of associated sequence. No *Bacteroides* pangenomes approached saturation even given the amount of sequence included in this study (average of 276, SD 93 additional pan-genes when moving from the 99th percentile to the whole set of reconstructed genomes), suggesting that even for common, well-studied organisms, a surprising amount of intra-species genomic diversity (and associated biochemical function) remains to be captured.

**Figure S6:**
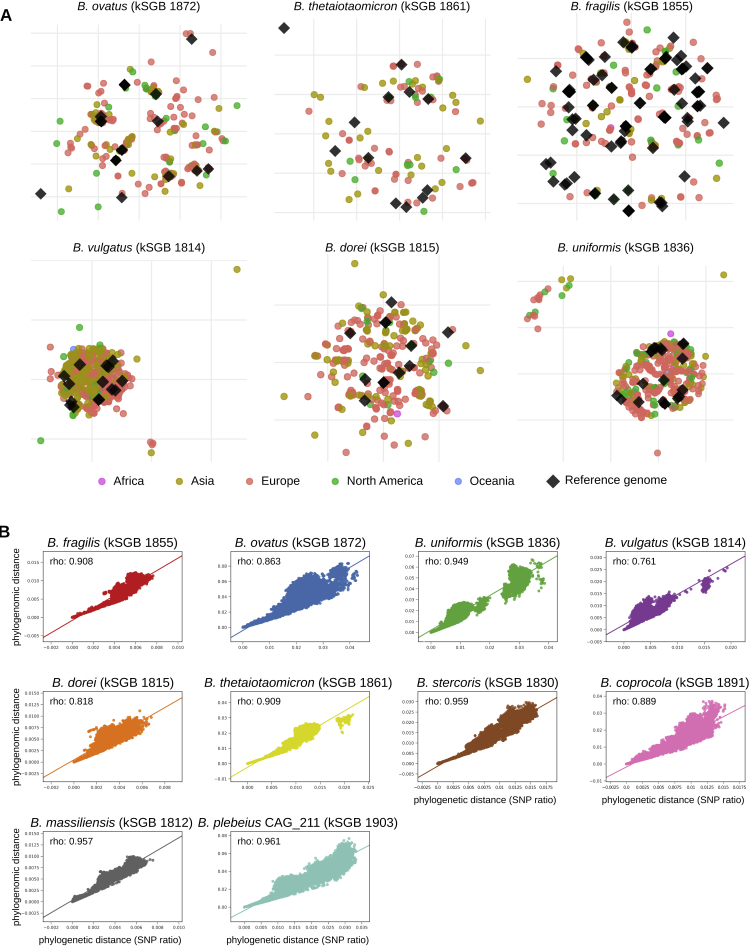
Genetic Diversity and Correlation between Genetic and Functional Similarity for Bacteroides Species, Related to Figure 4 (A) MDSs on intra-SGB genetic distances for *Bacteroides* species not reported in Figure 4C. (B) Scatterplots for the ten most prevalent *Bacteroides* kSGBs showing the relation between pairs of genomes measured as branch length distance on the core-genome-based phylogenetic tree (x axis) and as branch length on the hierarchical clustering built on the presence and absence of pan-genes (phylogenomic distance, y axis).

Most of the *Bacteroides* SGBs contained distinct subspecies clusters, and many of these subspecies include only genomes we reconstructed in this work (Figures 4C and S6A). Some of the most abundant *Bacteroides* species (including *B. stercoris* and *B. plebeius*) were only partially captured by isolate genomes, and the additional reconstructed genomes accounted for an average of 95.8%, SD 5.0% total branch length in the ten core-genome phylogenies. Considering that genetic sub-speciation is highly correlated with functional diversification (correlations > 0.8, p < 1e−50, Figure S6B), the reconstructed genomes thus uncover not only genetic diversity but also relevant functional diversity included in otherwise inaccessible *Bacteroides* subspecies.

### Some uSGBs and Subspecies Are Strongly Associated with Non-Westernized Populations

**Figure 5:**
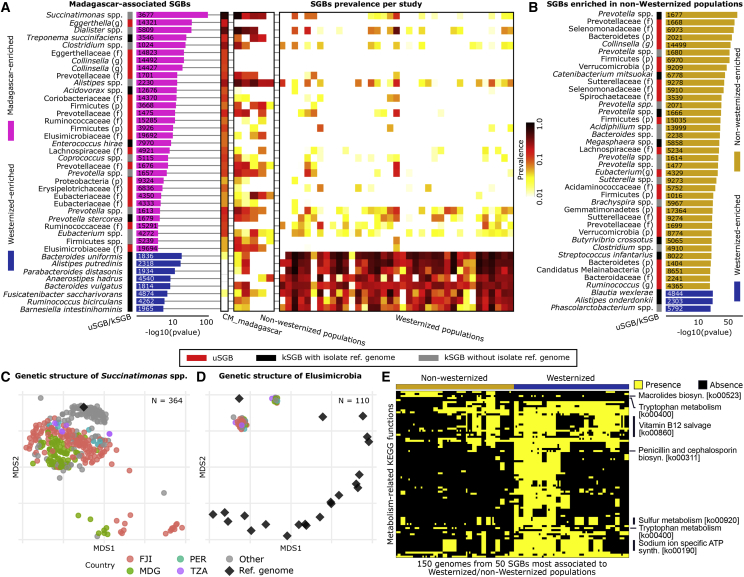
SGBs and Single Reconstructed Genomes Associated with Westernized and Non-Westernized Lifestyles (A) 49 total large (>10 genomes) SGBs were significantly enriched (Fisher's test) in the set of 112 Madagascar gut metagenomes sequenced for this study, and 20 were significantly depleted (Fisher's test) relative to Western gut microbiomes (complete results in Table S6). Most Madagascar-enriched SGBs are uSGBs or contain only isolate sequences that were themselves assembled from other metagenomes in other studies. (B) 232 total SGBs were differentially present with respect to the total set of non-Westernized populations, again with the 40 most significant—excluding those already reported in (A)—shown here (Fisher's test, complete results in Table S6). (C) The intra-SGB genetic structure of *Succinatimonas* spp., the bacterium most associated with non-Westernized lifestyles (multidimensional scaling [MDS] on percentage nucleotide distances between genomes). The few genomes assembled from Westernized countries are tightly clustering together, while strains from non-Westernized populations are distinct and not well represented by the only available co-assembled (but not cultivated) strain. (D) MDS of the two uSGBs (ID 19692 and ID 19694) enriched in the Madagascar cohort and available isolate genomes for the containing Elusimicrobia phylum (phylogeny in Figure S5A). The metagenomically assembled genomes in Elusimicrobia SGBs greatly diverge from the non-human-associated isolate genomes in the phylum. (E) Significant differences in functional potential between the 25 SGBs most strongly associated with Westernized and non-Westernized populations. We report the differential KEGG pathways (Fisher's test Bonferroni-corrected p < 0.05, full list in Table S6) whose components are found in the set of representative genomes for the 50 species (only three genomes per SGB).

To further assess the specificity of the unexplored uSGBs among global populations, we profiled the gut microbiomes of two rural communities with non-Western lifestyles from northeastern Madagascar (STAR Methods). The SGB profiles of the Madagascar population were profoundly different from that of Western-style populations (Figures 2C and 2D), with 49 of the 941 large (>10 genomes) SGBs highly enriched in this east-African population and 8 SGBs uniformly absent (20 total depleted SGBs, Fisher's test Bonferroni-corrected p < 0.05, Figure 5A, Table S6). An SGB that contains a previously co-assembled *Succinatimonas* sp. but no isolate genomes was the strongest association with the Madagascar population (Fisher's Bonferroni-corrected p = 8.2e−99), as well as with non-Westernized populations generally (p = 4.3e−244), across which it was successfully assembled in 55.9% of the samples (4.55% average and 56% maximum relative abundance) compared to only 1.6% in Westernized samples (3.34% average and 20.13% maximum relative abundance). The type strain of this genus (*Succinatimonas hippei*) was isolated from the gut of a healthy Japanese individual in 2010 (Morotomi et al., 2010) and is phylogenetically similar to isolates from poultry. The ability to degrade D-xylose is characteristic of the clade, a plant-sugar whose metabolism was previously reported as enriched in rural microbiomes (De Filippo et al., 2010). The phylogenetic structure of *Succinatimonas* SGB 3677 also suggests further specialization to specific host lifestyles at the subspecies level, with 99 of the 117 genomes from Westernized populations tightly clustering together and well separated from all 246 genomes from the five non-Westernized populations (Figures 5C and S5B). This SGB in the *Succinatimonas* genus shows a geographically consistent pattern of lifestyle association, resulting in dramatically different prevalences across the globe (p = 4.3e−244) as well as intra-species geographically specific genetic diversification.

The non-Westernized gut microbiome is overall enriched for uSGBs rather than kSGBs (Figures 5A and 5B), which was consistent despite the different protocols used in the considered studies (Table S6). These include several uSGBs in the Firmicutes and Actinobacteria phyla but also in less typically human-associated phyla such as the Elusimicrobia phylum. Two Elusimicrobia uSGBs were associated with the Madagascar (ID 19692 and ID 19694, Fisher's test p = 4.64e−11 and 9.76e−05, respectively) and non-Westernized gut microbiome (ID 19694, p = 1.52e−53) but showed 22% nucleotide divergence from the closest isolate genome (Figures 5D and S5A). 22 isolate genomes are available for this phylum, but they were typically recovered from termites and other insects (Herlemann et al., 2007) and were even more genetically distant from those we identified in humans (>30% nucleotide distance). While these divergent Elusimicrobia uSGBs populate the non-Westernized gut microbiome with some frequency (15.4% prevalence, 0.73% average relative abundance, Figure 5D), they are rarely found in Westernized individuals (0.31% prevalence).

*Bacteroides uniformis* was the strongest Westernized-lifestyle-associated bacterium (Figure 5B; Table S6), and 13 other *Bacteroides* species with a combined total of 10,992 genomes also showed the same trend (2.66% versus 0.86% prevalence and 5.77% Westernized versus 1.69% non-Westernized average abundance). With the exception of four unnamed low-prevalence *Bacteroides* SGBs (434 genomes in total), no species of this clade was significantly enriched in non-Westernized populations; instead, these were highly enriched in *Prevotella* species (12 kSGBs against no significant *Prevotella* kSGB in Westernized populations), as expected (De Filippo et al., 2010, Obregon-Tito et al., 2015). Several other known and relatively well-characterized species (including *Alistipes putredinis*, *Parabacteroides distasonis*, and *Akkermansia muciniphila*) were significantly associated with Westernized populations, in total accounting for >23 times more kSGBs than uSGBs. Conversely, among SGBs enriched in non-Westernized populations, uSGBs greatly outnumbered kSGBs (144 versus 63, Fisher's test p = 1.0e−23). This further confirms that populations with non-urbanized and traditional lifestyles have a more uncharacterized gut microbiome that is made more accessible to future characterization by these results.

Microbiome differentiation between lifestyles was also reflected at the functional level (Figure 5E; Tables S5 and S6). Sulfur energy metabolism (ko00920), vitamin B12 salvage (ko00860), and the sodium-ion-specific ATP synthase operon *ntp* (ko00190) were among the KEGG functional modules significantly enriched in Westernized microbiomes (Figure 5E). Other functions were present in both lifestyles but encoded by different enzymes and pathways. For example, both groups’ microbiomes encoded extensive antibiotic biosynthesis genes (Figure 5E), but while Westernized-enriched SGBs encoded the pathway for penicillin and cephalosporin biosynthesis (ko00311), non-Westernized-enriched SGBs more often carried genes for macrolide biosynthesis (ko00523). Similarly, genes for tryptophan metabolism were differently present in the two groups, with parts of the same pathway (ko00400) differentially present in Westernized and non-Westernized communities (Figure 5E). UniRef50 annotations of all genomes highlighted many additional differences (82,563 with Bonferroni-corrected p < 0.01, Table S5), spanning also fimbrial functions and degradation of complex pectins enriched in the non-Westernized microbiomes. These associations of microbial functional potential with population capture a wide range of potential diet, metabolic, genetic, and exposure differences (De Filippo et al., 2010, Yatsunenko et al., 2012) and suggest that there are multiple ways in which the gut microbiome adapts to the diversity of human hosts.

## Discussion

**Figure 6:**
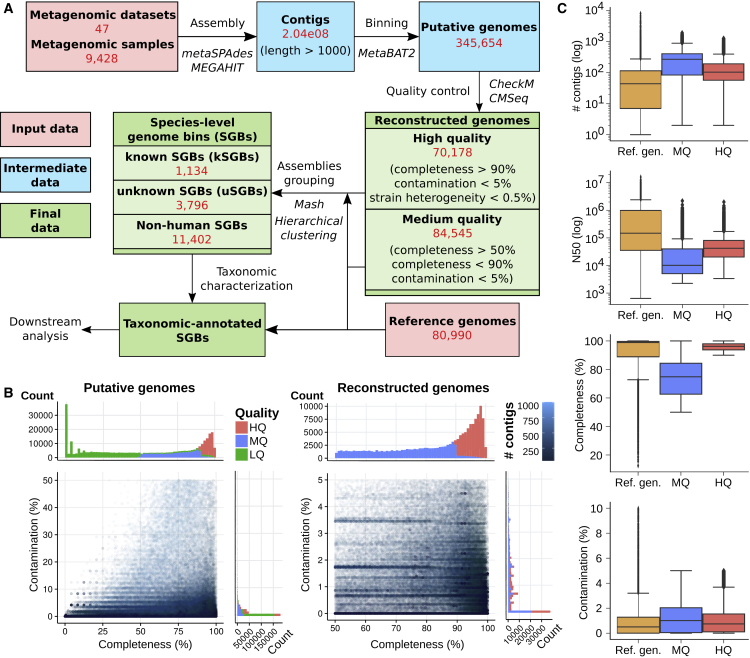
Methodology Overview and Quality Characteristics for the 154,723 Reconstructed Genomes (A) Overview of the overall strategy and datasets employed for the reconstruction of microbial genomes and their organizations in SGBs. (B) Completeness and contamination values estimated by CheckM are reported for LQ (low quality, completeness <50% or contamination >5%), MQ (completeness in the range [50%, 90%] and contamination <5%), and HQ (completeness >90%, contamination <5%, CMSeq strain heterogeneity <0.5%) genomes. LQ genomes are excluded from the rest of the analysis. (C) Comparisons between the genomes from UniRef/NCBI used as references and our reconstructed genomes.

**Figure 7:**
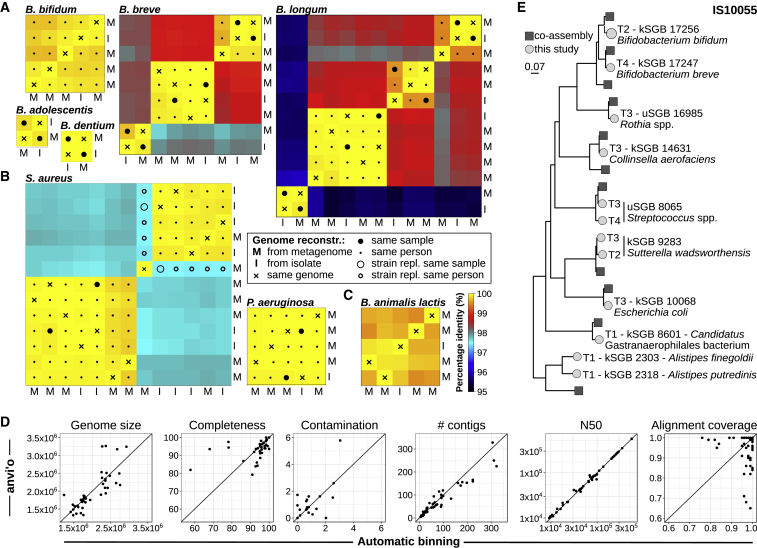
Quality of the Single-Sample Assembled Genomes against Multiple Alternative Genome Reconstruction Approaches (A) Percentage identity between genomes from isolates (I) and genomes we reconstructed from metagenomes (M) for five *Bifidobacterium* species from the FerrettiP_2018 dataset (Ferretti et al., 2018). We mark isolates and metagenomes coming from the same specimen (big filled circles) and coming from specimens of the same mother-infant pair (small filled circles). In all cases, our automatic pipeline reconstructs genomes from metagenomes that are almost identical to the genomes of the expected isolated strains. (B) The strains of *S. aureus* and *P. aeruginosa* isolated from three patients are almost perfectly matching the genomes reconstructed from sputum metagenomes sequenced at multiple time points. In the only case in which a *S. aureus* genome from a metagenome is not matching the strain isolated from a previous time point in the same patient, we verified with MLST typing that a clinical event of strain-replacement from ST45 to ST273 occurred. (C) In the dataset by Nielsen et al. (2014), we successfully recover at >99.5% identity the strain of a *B. animalis* subspecies lactis present in a commercial probiotic product that was consumed by the enrolled subjects, even if the probiotic strain was at low relative abundance in the stool microbiome (<0.3% on average [Nielsen et al., 2014]). (D) Comparison of the 46 manually curated genomes (using anvi’o) with automatically assembled (using metaSPAdes) and binned (using MetaBAT2) genomes. (E) Example comparison between the set of single-sample assembled genomes and co-assembled genomes for a time series (n = 5) of gut metagenomes from a newborn. Several genomes reconstructed with the two approaches have the same phylogenetic placement, with single-sample assembly retrieving the same (or a very closely related) genome at multiple time points, and both methods retrieving some unique genomes. This is an example of the comprehensive comparison performed in the STAR Methods and reported in Table S2 and Figure S7B.

**Figure S7:**
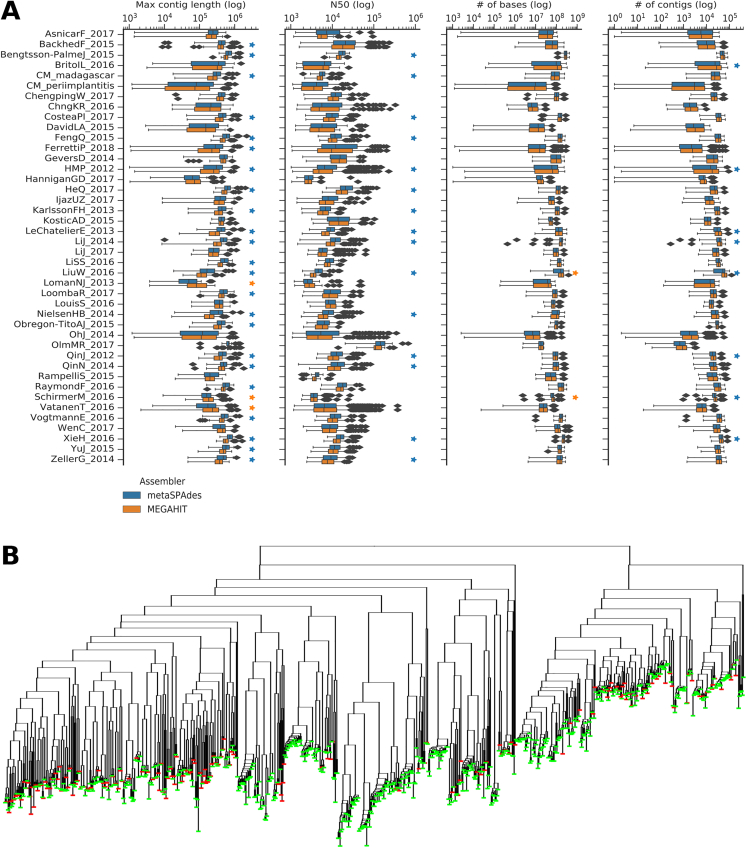
Comparison between MEGAHIt and metaSPAdes Assemblies and between Assembly and Co-assembly, Related to Figure 7 (A) Comparison between metaSPAdes and MEGAHIT assemblers across all the considered datasets confirms that metaSPAdes performs consistently better especially in recovering long contigs. Stars indicate statistically significance (Welch's t test, p < 0.05). (B) Phylogenetic tree built on the genomes of gut adult metagenomes from 25 women from the FerrettiP_2018 dataset showing comparison between the set of single-sample assembled genomes (in green) and co-assembled genomes (in red). Several genomes reconstructed with the two approaches have the same phylogenetic placement, with single-sample assembly retrieving a total of 605 genomes spanning 257 SGBs, while co-assembly retrieved 172 genomes.

This work expands the collection of microbial genomes associated with the human microbiome by more than doubling the current collections with over 150,000 newly reconstructed genomes, in the process recovering hidden functional and phylogenetic diversity associated with global populations (particularly those that are undersampled from non-Western lifestyles and non-gut areas, Figure 1E). More than 94% of metagenomic reads can now be mapped to the expanded genome catalog for half of the gut microbiomes, enabling a much more comprehensive profiling of these communities. The metagenomic-assembly strategies employed here (Li et al., 2015, Nurk et al., 2017) represent a scalable methodology for very large-scale integration of metagenomes (Figure 6) that we extensively validated (STAR Methods; Figures 7 and S7) and could be fruitfully applied to additional or non-human-associated metagenomes. The methods are also compatible with emerging technologies such as synthetic (Kuleshov et al., 2016) or single-molecule (Brown et al., 2017) long-read sequencing, which will further add to the diversity of microbial genomes. Finally, the study’s results themselves emphasize the phylogenetic and functional diversity that remains to be captured from rare organisms, especially for sample types other than stool, global human populations, and varied lifestyles for the human microbiome.

Even within the current data collection, a variety of results remain to be explored. Part of the metagenomic reads that could not be mapped against our extended bacterial and archaeal resource are likely coming from viral and eukaryotic genomes. For example, we found substantial amounts of viruses (>0.5% relative read depth in 101 samples for bacteriophages never found as prophages in reference bacterial genomes), of the intestinal eukaryotic parasite *Blastocystis* (>0.5% in 158 samples), and of the skin fungus *Malassezia* (>0.5% in 297 samples). Considering that *de novo* discovery of non-bacterial genomes is very challenging and should receive more attention in the future, eukaryotic microorganisms and viruses may thus account for some of the remaining unmappable sequences in these data (Figure 2). These results help to pinpoint microbes unique to a particular population, environment, or exposure, and most importantly, future work may then be able to more easily capture specific strains or microbial molecular mechanisms that are causal in microbiome-associated human health conditions.

## STAR★Methods

### Key Resources Table

**Table:** 

REAGENT or RESOURCE	SOURCE	IDENTIFIER
**Biological Samples**		

Stool samples from Madagascar cohort	Golden et al., 2017	N/A
Stool samples from Ethiopian cohort	This paper	N/A

**Critical Commercial Assays**		

PowerSoil DNA Isolation Kit	MoBio Laboratories Carlsbad, USA	Catalog No. 12888-50
NexteraXT DNA Library Preparation Kit	Illumina, California, USA	FC-131-1096

**Deposited Data**		

Raw sequencing data (Madagascar cohort)	This paper	NCBI-SRA BioProject: PRJNA485056
Raw sequencing data (Ethiopian cohort)	This paper	NCBI-SRA BioProject: PRJNA504891
Data for all genomes	This paper	http://segatalab.cibio.unitn.it/data/Pasolli_et_al.html
Representative genome for *Ca.* Cibiobacter qucibialis	This paper	DDBJ/ENA/GenBank accession SAUS00000000

**Software and Algorithms**		

metaSPAdes (version 3.10.1)	Nurk et al., 2017	https://github.com/ablab/spades/releases
MEGAHIT (version 1.1.1)	Li et al., 2015	https://github.com/voutcn/megahit
MetaBAT2 (version 2.12.1)	Kang et al., 2015	https://bitbucket.org/berkeleylab/metabat
CheckM (version 1.0.7)	Parks et al., 2015	https://github.com/Ecogenomics/CheckM
CMSeq (version 1.0.0)	This study	https://bitbucket.org/CibioCM/cmseq
Mash (version 2.0)	Ondov et al., 2016	https://github.com/marbl/Mash
MetaPhlAn2 (version 2.0)	Segata et al., 2012b, Truong et al., 2015	https://bitbucket.org/biobakery/metaphlan2
HUMANn2 (version 0.7.1)	Franzosa et al., 2018	https://bitbucket.org/biobakery/humann2/
Bowtie2 (version 2.2.9)	Langmead and Salzberg, 2012	https://github.com/BenLangmead/bowtie2
Prodigal (version 2.6.3)		https://github.com/hyattpd/Prodigal
Pyani (version 0.2.6)	Pritchard et al., 2016	https://github.com/widdowquinn/pyani
StrainPhlAn (version 2.0.0)	Truong et al., 2017	https://bitbucket.org/biobakery/metaphlan2
Anvi’o (version 4)	Eren et al., 2015	https://github.com/merenlab/anvio
BWA (version 0.7.17)	Li and Durbin, 2009	https://github.com/lh3/bwa
CONCOCT (version 0.5.0)	Alneberg et al., 2014	https://github.com/BinPro/CONCOCT
RPSBlast	Marchler-Bauer et al., 2003	ftp://ftp.ncbi.nih.gov/blast/executables/
PhyloPhlAn (version dev, 0.25)	Segata et al., 2013	https://bitbucket.org/nsegata/phylophlan
Diamond (version 0.9.9.110)	Buchfink et al., 2015	https://github.com/bbuchfink/diamond
mafft (version 7.310)	Katoh and Standley, 2013	https://github.com/The-Bioinformatics-Group/Albiorix/wiki/mafft
trimal (version 1.2rev59)	Capella-Gutiérrez et al., 2009	https://github.com/scapella/trimal
RAxML (version 8.1.15)	Stamatakis, 2014	https://github.com/stamatak/standard-RAxML
IQ-TREE (version 1.6.6)	Nguyen et al., 2015	https://github.com/Cibiv/IQ-TREE
Roary (version 3.8)	Page et al., 2015	https://github.com/sanger-pathogens/Roary
blastn (version 2.6.0+)	Altschul et al., 1990	ftp://ftp.ncbi.nlm.nih.gov/blast/executables/blast
FastTree (version 2.1.9)	Price et al., 2010	https://github.com/PavelTorgashov/FastTree
ecodist R package	Goslee and Urban, 2007	https://github.com/cran/ecodist
GraPhlAn (version 1.1.3)	Asnicar et al., 2015	https://bitbucket.org/nsegata/graphlan/
FigTree (version 1.4.3)	N/A	http://tree.bio.ed.ac.uk/software/figtree/
Prokka (version 1.12)	Seemann, 2014	https://github.com/tseemann/prokka
EggNOG mapper (version 1.0.3)	Huerta-Cepas et al., 2017	https://github.com/jhcepas/eggnog-mapper
HMM	Eddy, 2011	https://github.com/guyz/HMM
Barrnap (version 0.9)	N/A	https://github.com/tseemann/barrnap
RDP (version 2.11)	Cole et al., 2014, Wang et al., 2007	https://github.com/rdpstaff/classifier

**Other**		

curatedMetagenomicData	Pasolli et al., 2017	https://github.com/waldronlab/curatedMetagenomicData
UniProt	The UniProt Consortium, 2017	https://github.com/ebi-uniprot
NCBI GenBank database	NCBI Resource Coordinators, 2013	https://www.ncbi.nlm.nih.gov/genbank/
RefSeq (viral genomes and plasmids)	Brister et al., 2015, O’Leary et al., 2016	https://www.ncbi.nlm.nih.gov/refseq/

### Contact for reagent and resource sharing

Further information and requests for resources, reagents, and software should be directed to and will be fulfilled by the Lead Contact, Nicola Segata (nicola.segata@unitn.it).

### Experimental model and subject details

Subjects enrolled in our study are adults from the Madagascar and Ethiopian non-Westernized cohorts described in the Methods below. Ethical approvals were given by the Madagascar Ministry of Health and the Office for the Protection of Human Subjects at the Harvard T.H. Chan School of Public Health, protocol #22826 for the Madagascar cohort, and by the Research Ethics Committee of the Valencia University (reference number: H1484811493170) and also by the Ethics Committee of the Consejo Superior de Investigaciones Cientìficas (Madrid, Spain), number 058/2018, for the Ethiopia cohort. Informed consent was obtained for all individuals.

### Method details

#### Overview of the approach

Our approach to reconstruct bacterial and archaeal genomes from the human microbiome (Figure 6A) exploits metagenomic single-sample assembly, contig binning, and species-level inter-sample genome grouping at the scale of the many thousands of metagenomes now available in public repositories.

In brief, we first collected and curated a metagenomic resource comprising a total of 9,428 metagenomes (from public resources and samples sequenced in this study, see below) and then applied metagenomic assembly - metaSPAdes (Nurk et al., 2017) or MEGAHIT (Li et al., 2015) - to each sample separately. Each metagenomic assembly was then quality controlled for minimum length and the 204M contigs were subjected to sample-specific contig binning based on tetranucleotide frequency and contig abundance using MetaBAT2 (Kang et al., 2015) resulting in over 345,000 putative genome bins (Figure 6A). Genome bins were then strictly quality controlled to identify reconstructed genomes with quality at least comparable with the typical quality of isolate genome sequencing. By controlling genome completeness and contamination using CheckM (Parks et al., 2015) and strain heterogeneity with the CMSeq pipeline described below, we identified 70,178 high-quality genomes and 84,545 additional MQ genomes (Figure 6A).

The 154,723 reconstructed genomes and the 80,990 reference genomes retrieved from public repositories (see below) were then clustered based on whole-genome nucleotide similarity estimation using Mash (Ondov et al., 2016). The cutoff on the hierarchical clustering was tuned based on the intra- and inter-species diversity of the confidently taxonomically labeled subset of the 80,990 reference genomes resulting in species-level genome bins (SGBs) spanning ∼5% genetic diversity, as independently proposed elsewhere (Jain et al., 2018). Overall we obtained 16,332 SGBs that were further divided in known SGBs (kSGB) that contain at least one reference genome, unknown SGBs (uSGBs) without any reference genomes, and non-human SGBs containing only reference genomes and no genomes reconstructed from our assembly of the human microbiome (Figure 6A). The kSGBs were then taxonomically labeled with the species label (if available) of the reference genome(s) present in the bin, whereas uSGBs were assigned the phylum of their closest reference genome, and to a genus-level and family-level annotation when possible.

#### Meta-analyzed publicly available metagenomic datasets

We collected publically available metagenomic samples from 46 different studies, totaling 9,316 metagenomes and 4.1e11 Illumina reads. Overall, the samples cover 31 countries: USA (1,431 samples), China (1,342), Israel (956), Sweden (600) and Denmark (580) are the 5 most represented. The metagenomes were sampled from 5 major body sites: 7,783 samples from the gut (stool samples), 783 from the oral cavity, 503 from the skin (including 93 samples from anterior nares), 88 from the vagina, and 9 from maternal milk (excluded for visualization from the figures). Samples from adults (19 to 65 years of age) account for 6,615 samples, but all age categories are covered with 1,098 newborns (< 1 year of age), 465 children (age ≥1 year and <12 years), 216 school-age individuals (age ≥12 and <19 years), and 876 from adults and seniors (age ≥19 and >65 years; merged with the class “adult” in Figure 1). Despite manual curation efforts, 46 samples from public repositories used here still miss the metadata for age category. All these and other manually-curated metadata fields are available in Table S1 and are included in the *curatedMetagenomicData* package (Pasolli et al., 2017) together with all the taxonomic (Segata et al., 2012b, Truong et al., 2015) and functional potential profiles (Franzosa et al., 2018) of the microbial species with available reference genomes. To cross-validate the results on the raw-reads mappability, we also retrieved 384 additional metagenomes not used to reconstruct the SGBs. Specifically, we considered 303 Westernized gut metagenomes, 52 Westernized oral metagenomes and 29 non-Westernized oral metagenomes as reported in Table S1.

#### Enrollment of participants from non-Westernized populations from Madagascar and Ethiopia

We enrolled, sampled, and sequenced the gut microbiome of individuals from the Madagascar Health and Environmental Research (MAHERY) study cohort that was set up in 2004 in a remote rainforest region in north-eastern Madagascar to study the impact of environmental change on human health (Golden et al., 2017). The cohort includes local people (Betsimisaraka and Tsimihety ethnicity) whose diet relies heavily on self-grown rice and wild plants and meats. Samples were collected between January 2013 and May 2014 from two subsistence communities (A and B) adjacent to the Makira Natural Park, approximately 10 km away from each other. A subset of the households in the two communities were randomly selected to be enrolled in the study (95 households out of 160 in Community A and 57 households out of 157 in Community B), for a total of 719 individuals < 74 years old. Enrolled people were subjected to clinical visits and questionnaires about dietary intake, and were asked to collect biological samples (fingernails, blood, faeces) to assess health and nutritional status. The samples considered in this study were collected from a total of 112 healthy volunteers (54 females and 58 males, Table S1). The gut microbiome of five female individuals were also sampled from a previously established cohort in Gimbichu (Ethiopia, Oromia Region).

#### Sample collection of non-Westernized cohorts

Faecal samples from the Madagascar cohort were self-collected in sterile polypropylene screw cap collection tubes (Sarstedt) after defecation on the waxy side of a banana leaf, and returned to the local research team within five hours of collection (Golden et al., 2017). Three ml of 97% ethanol were added to stabilize samples before storing them at −23°C within 14 days of collection. Samples were then shipped on dry ice to the USA to be stored at −80°C. Faecal samples from Ethiopian individuals were collected in REAL MiniSystem “Total - fix” (Durviz S.L., Valencia, Spain) and kept frozen at −80°C.

#### DNA extraction and sequencing

DNA was extracted with the PowerSoil DNA Isolation Kit (MoBio Laboratories) after pre-heating to 65°C for 10 min and to 95°C for 10 min (HMP Consortium, 2012). Libraries were prepared with the NexteraXT DNA Library Preparation Kit (Illumina) and sequenced on the HiSeq2500 machine (Illumina). The metadata for this cohort are available in Table S1 and are included in the *curatedMetagenomicData* package together with the taxonomic and functional potential profiles of the species with available reference genomes. We sequenced the 117 samples for a total of 593.9 Gb (5.3 Gb average per sample after quality control, 3.87 Gb standard deviation, Table S1). The raw reads were submitted to the NCBI-SRA archive and are available under the BioProjects PRJNA485056 (Madagascar cohort) and PRJNA504891 (Ethiopian cohort).

#### Description of the non-Westernized cohorts

Westernization and urbanization are complex processes that occurred during the last few centuries involving profound lifestyle changes compared to populations prior to the modern era. These changes include increased hygiene and sanitized environments, introduction and large availability of antibiotics and other drugs, switch toward a high-calorie high-fat dietary regimes and toward processed sterilized food, enhanced exposure to xenobiotics and pollutants, reduced contact with wildlife and domesticated animals, and transition from autarchic food production systems to a controlled food chain in a global economy. All these factors are thought to have dramatic effects on the human microbiome that co-evolved with our our body for hundred thousands of years in non-Westernized conditions. In this work, we adopt the terms “Westernized” and “non-Westernized” as umbrella terms to depict populations that differ by at least the majority of the above factors even though this definition comprises very heterogeneous populations.

In addition to the sequenced Madagascar cohort (above), 480 additional samples were annotated as “non-Westernized” from a total of 5 studies spanning 4 populations. These were a traditional Fijian population (Brito et al., 2016) (172 stool samples and 140 saliva samples), the hunter-gatherer Hadza population (Tanzania) from two different studies (Rampelli et al., 2015, Smits et al., 2017) (67 stool samples in total), the traditional agro-pastoral Mongolian population (Liu et al., 2016) (65 stool samples), and a Peruvian rural community (Obregon-Tito et al., 2015) (36 stool samples). With the Madagascar cohort, this work thus considers a total of 592 non-Westernized compared with 8,836 Westernized samples.

#### Isolate genomes and available metagenomic assemblies used as references

We considered the whole set of 17,607 microbial species (16,959 bacteria, 648 archaea) available as of March 2018 in the UniProt portal (The UniProt Consortium, 2017) for which at least one proteome (the set of coding sequences associated with the genome) is available. Quality control performed by UniProt to retain the proteomes and the associated genomes include the availability of a set of annotated coding sequences and the check that the number of coding sequences is statistically consistent with the one of proteomes of neighboring species. We then considered all the available annotated genomes for these species and downloaded them from the NCBI GenBank database (NCBI Resource Coordinators, 2013) obtaining a total of 80,853 genomes. This large genome set comprises both complete (12%) and draft (88%) genomes, and it is the largest set of microbial isolate genomes with taxonomic assignments and quality-controlled sequences available as of March 2018. Draft genomes include also metagenomic species that are explicitly labeled with the “MAG” abbreviation (n = 37) and co-abundance gene groups metagenomic assemblies (CAGs, n = 377) (Nielsen et al., 2014). We further added this genome set to the 137 isolate genomes collected in (Browne et al., 2016) for a total of 80,990 considered as reference genomes. We refer to this set of 80,990 as “isolate genomes” for brevity, but they also comprise previous metagenomic assembly as mentioned above. To further expand the set of reference genomes we also considered all the 159,803 assemblies available in NCBI as of September 2018.

#### Metagenomic assembly and contig binning

Each of the 9,428 samples were processed with the standard quality-control employed by metaSPAdes (Nurk et al., 2017) which includes the read corrector BayesHammer (Nikolenko et al., 2013) and then independently subjected to *de-novo* metagenomic assembly through metaSPAdes (Nurk et al., 2017) (version 3.10.1; default parameters), which exhibited the best accuracies in recent comparisons among metagenomic assemblers (Forouzan et al., 2018, van der Walt et al., 2017). Samples that failed to be processed due to memory requirements (>1Tb of RAM), and samples with only unpaired reads, were assembled through MEGAHIT (Li et al., 2015) (version 1.1.1; default parameters). An extended comparison between metaSPAdes and MEGAHIT assemblers across all the datasets considered in this study confirmed that metaSPAdes performs consistently better especially in recovering long contigs (Figure S7A). Contigs shorter than 1,000 nt were discarded from further processing. This resulted in 2.04e8 different contigs for a total length of 8.67e11 nt. Reads were mapped to contigs using Bowtie2 (Langmead and Salzberg, 2012) (version 2.2.9; option ‘--very-sensitive-local’) and the mapping output was used for contig binning through MetaBAT2 (Kang et al., 2015) (version 2.12.1; option ‘-m 1500’), which showed good performance in comparison with other binning methods (Meyer et al., 2018). MetaBAT2 achieved the best performances among single-sample binning tools also in the evaluation performed in the Metawrap paper (Uritskiy et al., 2018), a recent tool for multiple binning. The multiple binning approach looks promising, although lack of independent validation and high computational requirements make it infeasible to be used in the large-scale scenario exploited in this paper at this stage. The procedure of binning through MetaBAT2 generated 345,654 bins (i.e., putative genomes) for a total length of 6.55e11 nt indicating that 75% of the assembled contigs were grouped into bins.

The relative abundance of each reconstructed genome in the 9,428 metagenomes was calculated from the alignments of the raw reads against the assemblies of the same sample (performed using BowTie2 as reported above). This avoids spurious read assignments (i.e., reads mapping sufficiently well against more than one genome in the same or different species). Indeed, as a direct consequence of the assembly-based approach, it is very rare (< 0.01%) that a read can be assigned to more than one contig assembled from a metagenome containing the read itself. Thus, the relative genome abundance in each sample was defined as the number of reads aligning to each contig of the genome normalized by the total number of reads in the sample. Only primary alignments with alignment length ≥50 nt and edit-distance with respect to the contig ≤2 nt were considered. Abundances at SGB level in each sample were computed as the sum of the abundances of the reconstructed strains belonging to the same SGB.

#### Quality control of metagenomic assemblies

Putative genomes were subjected to quality control to generate the final set of reconstructed draft genomes. Three main measures were taken into account: i) completeness; ii) contamination; and iii) strain heterogeneity. Completeness and contamination were estimated using CheckM (Parks et al., 2015) (version 1.0.7; lineage specific workflow), while strain heterogeneity was estimated through a strategy we developed to identify assemblies resulting from strain mixtures even when the strains were very closely related. Following this procedure, reads were mapped against the reconstructed genomes from the same sample using Bowtie2 (Langmead and Salzberg, 2012) (version 2.2.9; option ‘--very-sensitive-local’) and dominant and non-dominant alleles were determined over all protein coding nucleotides. We only considered base calls with a PHRED quality score of at least 30 and only those positions with a coverage of at least 10x. We considered a position as non-polymorphic if the dominant allele frequency was >80%. In order to calculate the polymorphic rate, we then considered only polymorphic positions corresponding to non-synonymous mutations. Validation experiments performed by mixing simulated metagenomic sequencing (with Illumina error models) of 5 randomly selected pairs of strains from each of the the 10 *Bacteroides* species of Figure 4 at decreasing dominant strain frequency (and thus higher nucleotide-level heterogeneity) confirmed that this approach reflects indeed the expected level of strain mixture. The strain heterogeneity estimation tool is available at https://bitbucket.org/CibioCM/cmseq.

Based on these quality estimated and on recent guidelines (Bowers et al., 2017), we selected as medium-quality (MQ) genomes those having completeness >50% and contamination <5% resulting in a total of 154,723 microbial genomes. Stricter quality control reduced the set of near-complete, high-quality (HQ) genomes to 70,178 with completeness >90% and no evidence of strong intra-sample strain heterogeneity (<0.5% polymorphic positions). The strain heterogeneity threshold removed 3,653 reconstructed genomes (5.2%) of otherwise HQ genomes, and we verified that these genomes tended to have higher CheckM contamination (although always below the recommended 5% threshold) with a median of 0.74% against 1.56% (p < 1e-50). This provides an additional indication that the CMSeq heterogeneity score helps in controlling strain mixtures and contaminations.

We evaluated the presence of plasmids and viruses within reference genomes and reconstructed SGBs by mapping the 13,924 plasmids and 10,529 viruses in RefSeq against the 80,990 reference genomes and the 154,723 genomes in the SGBs with BLAST (Altschul et al., 1990). We filtered alignments shorter than 500 nucleotides and with less than 80% identity. A plasmid or virus was considered to be present if at least 50% of its sequence was covered by any genome or SGBs in our catalog. We found that 37% of the fully sequenced plasmids in the RefSeq repository were represented in the reconstructed genomes (95% in the available reference genomes). The 16S rRNA sequences in the SGB genomes were searched with Barrnap 0.9 (default parameters). The 16S rRNA taxonomy (Table S4) was inferred with RDP rRNA classifier version 2.11 (Cole et al., 2014, Wang et al., 2007) (default parameters), only on predicted rRNA sequences longer than 500 nucleotides. We set RDP’s minimum confidence threshold to call for each taxonomic level at 75%. Although we confirmed that the 16S rRNA gene is challenging to be recovered by metagenomic assembly (it was recovered in only 7.43% of the reconstructed genomes), the search for the most 400 conserved coding genes from PhyloPhlAn (Segata et al., 2013) in the reconstructed genomes and isolate sequencing available for the 9 largest SGBs and the 10 *Bacteroides* SGBs of Figure 4, confirmed that cross-species conservation of genes is not an issue for metagenomic assembly. Metagenomically reconstructed genomes recovered more PhyloPhlAn markers in 10 cases and less markers in 9 cases, and all comparisons were within 5% average differences.

#### Validation of the pipeline for genome reconstruction from metagenomics using isolate sequencing and manually curated genomes

Genomes reconstructed from metagenomes were compared with the ones of isolates obtained from the same sample, or from samples obtained from the same individual at earlier or later time points (Figures 7A–7C; Table S2). We compared 18 isolates with 36 genomes reconstructed from metagenomes from 8 different bacterial species. Compared samples included sputum from cystic fibrosis patients (Manara et al., 2018), stool and breast milk samples from mother-infant pairs (Ferretti et al., 2018), and feces of adults consuming fermented milk product containing a probiotic strain (Nielsen et al., 2014).

Comparison between the genome reconstructed from the automatic pipeline and the one from isolate was done by computing the average nucleotide identity (ANI) and the corresponding alignment coverage using the pyani tool (Pritchard et al., 2016) (version 0.2.6; option ‘-m ANIb’). Results showed that in all cases the genomes reconstructed from metagenomes with our automatic pipeline were almost identical to the genome of the expected isolated strains. For the only case in which this was not true (*S. aureus* isolate MF093 and paired metagenome CM_cf__CF_FIFC009SS_t3M17__bin.3), we verified with MLST typing (both from assembled and unassembled reads) and with StrainPhlAn (Truong et al., 2017) that a clinical event of strain-replacement from ST45 to ST273 occurred.

A similar analysis was conducted to compare the genomes reconstructed using our fully automated pipeline with the ones obtained through manual curation using anvi’o (Eren et al., 2015) (Figure 7D; Table S2). Manually curated genomes were generated starting from the same set of unbinned contigs. A total of 50 metagenomes from the database considered in this study were randomly selected and assigned to six groups of students that were previously trained for the task of manual curation of contig binning by guided execution and discussion of the available anvi’o tutorials followed by curation of several example metagenomes common to all groups. Each group was asked to bin contigs for the strain with the highest reconstruction quality in the sample. This resulted in 46 manually-curated reconstructed genomes. Our automatic procedure recovered a genome closely matching (>99.5% whole genome genetic identity) the manually-curated one in all 46 cases. The comparison between genomes was done by computing the ANI score through the pyani tool and the results are reported in Figure 7D and Table S2.

#### Evaluation of single-sample assemblies against co-assembly and co-binning methods

In order to provide a comparison to the single sample strategy employed here, we co-assembled and co-binned a subset of the data where multiple samples from the same individual were available. Samples were taken from two studies: the already described investigation of the microbiome of newborns and of their mothers (Ferretti et al., 2018), and a study considering fecal microbiome time series for adults (Costea et al., 2017). From the first, we selected 22 infants for which at least 3 fecal samples taken during the first four months post-partum were available (maximum 5, median 4). We also co-assembled 21 fecal samples from the mothers from the same study to provide a comparison against cross-sectional co-assembly. Somewhat longer fecal time series were available from the second study, from which we selected four individuals with a number of time points between eight and ten (Costea et al., 2017). This gave us a total of 26 longitudinal time series from the same individual and one cross-sectional study (21 individuals) each of which we co-assembled using MEGAHIT (Li et al., 2015) with default parameters except for the kmer-list set to (21,31,..,99). The assembled contigs were then cut into 10kbp fragments and the reads from each sample within the time series (or mother in the cross-sectional study) were then mapped back onto the contig fragments using BWA and a per sample depth of coverage was calculated (Li and Durbin, 2009). The contig fragments were then clustered using the CONCOCT algorithm (default parameters) which combines both tetramer composition and coverage in a Gaussian mixture model after a PCA based dimensionality reduction (Alneberg et al., 2014). Following clustering, a consensus cluster assignment across fragments was given to each contig to assign clusters based on the original co-assembly.

We called ORFs on the co-assembled contigs and assigned COGs (Tatusov et al., 2003) using RPSBlast. The same procedure was applied to the genomes reconstructed by single sample assembly from the same set of samples used in each co-assembly. We then selected only those reconstructed genomes from both studies that possessed more than 75% of a panel of 36 single copy core genes in single copy (Alneberg et al., 2014). To remove redundancy across reconstructed genomes from the single sample clustering (i.e., same genome reconstructed at multiple time points from the same individual), and to determine the intersection of genomes between the two approaches, we then performed a hierarchical average linkage clustering of all the genomes from both methods and clustered at 1% nucleotide identity on the core gene panel. The results of such procedure are given in Table S2. We then also evaluated the genomes obtained by the co-assembly by computing CheckM completeness, CheckM contamination, and CMSeq heterogeneity as described for the single-assembly reconstructed genomes. Co-assembled genomes were then assigned to the HQ or MQ category with the same thresholds used for the single-assembly reconstructed genomes. The number of HQ and MQ genomes obtained with the two approaches was then compared, and additional genome quality metrics such as genome length, N50, completeness estimate, and contamination estimates were considered. For the genomes obtained by single-sample assembly, the grouping into SGBs was used to compare the number of distinct species obtained compared to the co-assembly approach. This second set of evaluations is also reported in Table S2.

For the short infant time series, the increase in number of genomes obtained by co-assembling and co-clustering was typically modest after collapsing closely related strains from single-genome assembly (median increase of 3% for the 36-core gene based evaluation - Table S2, 6.87% for the CheckM-based evaluation with thresholds for HQ genomes - Table S2). Without removal of closely related strains, single-genome assembly recovered more genomes (12% HQ genomes, 50% MQ genomes) because the same strains (or closely related ones) were recovered at multiple time points (Figures 7E and S7B).

The improvement for the co-assembly approach was more clear from the second study where at least eight time points were available (median increase 31% - Table S2). Across all the considered individuals there was a weak correlation between increase in the number of reconstructed genomes obtained from co-clustering and sample number (p = 0.08). We conclude that co-assembling and co-binning of gut metagenomes requires a moderate number of samples (more than 5) to achieve substantial improvements. The co-assembly of mothers yielded an increase of 3% in the number of HQ genomes (after merging single-sample assemblies into 99% identity genome bins) when using the 36 single-copy genes for quality control (Table S2), and a decrease from 124 to 88 HQ SGB-grouped genomes when using >90% CheckM completeness and < 5% CheckM contamination thresholds (Table S2). Other genome quality statistics were very similar between the two approaches with however the co-assembly method showing slightly more contamination (1.7% against 0.9% for HQ genomes, Table S2). Overall, this suggests that large scale co-assembly may at best offer limited improvement in terms of overall recovered diversity.

It is of note that the co-assembly approach can reconstruct only one bin per species or subspecies (Figures 7E and S7B) and on a large cross-sectional database such as the one considered in this study, this would effectively be a composite population-level genome incorporating both variation in single-nucleotide variants on core genes and variation in accessory genes. It is possible to resolve this variation on co-assemblies via single-nucleotide variant calling (Quince et al., 2017b, Truong et al., 2017) and when this is followed by deconvolution of haplotypes across samples as employed in the DESMAN pipeline (Quince et al., 2017b) this does allow the reconstruction of whole-genome haplotypes and assignment of accessory genes to specific strains. However, when most species are present in a single dominant strain as it is the case in the human microbiome (Truong et al., 2017), directly assembling strains from individual samples is a more straightforward strategy that both avoids the deconvolution step and uncertainties associated with variant calling from mapped reads. It is therefore more suitable for the very large scale analyses considered here where the aim is to generate a small number of HQ strains from each sample to provide the most comprehensive picture of overall diversity in the human gut.

The general conclusion of this comparison is thus that co-assembly and co-binning approaches would be useful for retrieving substantially more genomes in relatively long (>5) subject-specific time series, whereas the potential advantage of retrieving more low-abundance species in a cross-sectional co-assembly is overcome by the disadvantage of having to use more complex approaches such as DESMAN to resolve the strain variation. That is perhaps more appropriate where the aim is to extract as much information as possible from a single study rather than to produce a single comprehensive high fidelity strain catalog. Because time series comprising more than 5 samples from the same subject and body site are very rare in the available cohorts (only 70 individuals - i.e., 1.0% - in our database), co-assembly is not considered in the present work as it would not provide advantages.

#### Grouping of metagenomic assemblies into species-level genome bins

The 154,723 reconstructed genomes, in addition to the 80,990 reference genomes, were organized into species-level genome bins (SGBs). We applied an all-versus-all genetic distance quantification (nucleotide identity) on the total of 235,713 genomes using Mash (Ondov et al., 2016) (version 2.0; option “-s 1e4” for sketching) followed by hierarchical clustering with average linkage (using the fastcluster Python library).

The cutoff on the resulting dendrogram to define species-level genome bins (SGBs) was selected based on the intra- and inter-species diversity of the confidently taxonomically labeled subset of the 80,990 reference genomes. Microbial species labels for the genomes were inferred from the taxonomic label provided by NCBI GenBank in association with the genomes, and excluding all genomes containing ambiguous terms in the species name (i.e., “_sp,” “archaeon,” “bacterium,” or “candidatus”). This resulted in a total of 61,198 genomes spanning 5,494 named species.

With this labeling, the optimal dendrogram cutoff threshold to defined species-level genome bins (SGBs) was then chosen by taking into account two competing criteria (Figure S2C): i) minimization of the over-clustering error (Figure S2C**-i**) to avoid that genomes from the same species fall into different SGBs; ii) minimization of the under-clustering error (Figure S2C**-ii**) to prevent that genomes from different species fall into the same SGB. The two criteria were computed across all available species and cutoff choice, normalized by the total number of available genomes, and summed up to get the value to minimize. Results showed a minimization of the error for a threshold equal to 0.05 (Figure S2C**-iii**), which was thus adopted to cut the dendrogram and generate SGBs spanning ∼5% genetic diversity. A similar 5% genetic diversity range to define species boundaries was independently proposed and validated elsewhere (Jain et al., 2018), thus serving as a reasonable compromise despite the wide diversity of genomic similarities within existing defined species. This threshold was also confirmed by considering only prevalent species (>10 genomes) representing more studied and validated species, and by subsampling to a maximum of 10 genomes per species in order to avoid biases due to the different number of available genomes in existing species.

The resulting SGBs were further refined in order to prevent that same-species genomes were split into multiple SGBs due to inaccurate estimation of Mash for incomplete draft genomes. First i) a representative genome was selected for each SGB (Table S4). This was done by ranking genomes based on five metrics: completeness (in decreasing order), contamination (increasing), coverage (decreasing), strain heterogeneity (increasing), N50 (decreasing). The representative genome was selected as the one minimizing the sum of the five ranks. Then ii) the closest SGB was identified for each SGB based on the distances among representatives and iii) a more accurate ANI score was computed between each pair of close SGBs using the pyani tool (Pritchard et al., 2016) (version 0.2.6; option ‘-m ANIb’). Finally, iv) pairs of SGBs having an ANI score >95% were merged into a single SGB and v) the process was iterated until no more mergings were obtained. This merging operation reduced the number of SGBs of 3% resulting in a total of 16,332 distinct SGBs.

The obtained SGBs can be subdivided into 3 main groups (Figure 1B): i) the set of 1,134 known SGBs (kSGBs) that contain at least one reconstructed and one reference genome (the “unknownness” score for an SGB represents the number of reconstructed genomes with respect to the total number of genomes belonging to the SGB); ii) the set of 3,796 unknown SGBs (uSGBs) that contain at least one reconstructed genome, but no reference genomes from isolate sequencing or publicly available metagenomic assemblies; (iii) the set of 11,402 non-human SGBs, which contain at least one reference genome, but no reconstructed genomes. Results reported in the manuscript that involved computation of the ANI score for a number of genomes minor than 100 were done using pyani (Pritchard et al., 2016), while in the other cases we relied on the Mash (Ondov et al., 2016) estimates.

The kSGBs were taxonomically labeled with the species label associated with the reference genome(s) present in the bin, considering the most common species label if multiple reference genomes with different assigned species are present (Table S4). For uSGBs, no reference genomes were present in the species-level bins by definition, and we thus provided an assignment at higher taxonomic level. The same procedure used to find the optimal genomic divergence cutoff to define SGBs described above and in Figures S2A–S2C was adopted to define genus-level and family-level genomic divergence. Results showed a minimization of the error for a threshold equal to 0.15 and 0.30 for genus-level and family-level bins, respectively (Figure S2D), which was thus adopted to generate genus-level genome bins (GGBs) and family-level genome bins (FGBs). Although we are not proposing to modify the underlying taxonomy based on GGBs and FGBs, this additional clustering allowed us to give confident genus-level assignments to the 1,472 uSGBs falling in a GGB and a family label to 1,383 additional uSGBs falling in a FGB. Higher taxonomic levels are challenging to recapitulate by whole-genome clustering because of limitations in whole-genome nucleotide similarity quantification at large phylogenetic divergences, and we thus decided to maintain the remaining 941 unlabeled uSGB taxonomically unassigned. Nevertheless, for each SGB we report the full taxonomy of the closest matching genome and the whole-genetic distant from it to provide a genomic context for all SGBs (Table S4). The information about the closest labeled genome for the 941 uSGBs not assigned to a GGB or a FGB is used to assign them a phylum-level taxonomy in the text and in the figures. Finally, a taxonomic estimation based on 16S rRNA sequences was provided for 135 of these 941 uSGBs following the procedure described above in the section “Quality control of metagenomic assemblies“.

The set of 159,803 genomes available at the NCBI as of September 2018 was also considered to verify that our set of reconstructed genomes adds a substantial amount of unknown diversity. Indeed, we found that there were only 644 genomes that belong to uSGBs (1.9% of the set of reconstructed genomes in our uSGBs) using the same 5% whole-genome nucleotide divergence threshold described above. These 644 genomes, along with future updates, are added to the final resource available for download and we will continue integrating our resource with additional metagenomic assemblies and reference genomes that become available.

#### Reconstruction of the human-microbiome phylogenetic structure

The phylogenetic analyses were performed with PhyloPhlAn (Segata et al., 2013) using the “dev” branch of the repository as of end of June 2018 (https://bitbucket.org/nsegata/phylophlan/overview).

The phylogeny in Figure 1A was built using the 400 universal PhyloPhlAn markers with the following options: “--diversity high --accurate --min_num_markers 80.” For the internal steps the following tools with their set of parameters were used:

• diamond (version v0.9.9.110, (Buchfink et al., 2015)) with parameters: “blastx --quiet --threads 1 --outfmt 6 --more-sensitive --id 50 --max-hsps 35 -k 0” and with parameters: “blastp --quiet --threads 1 --outfmt 6 --more-sensitive --id 50 --max-hsps 35 -k 0”;
• mafft (version v7.310, (Katoh and Standley, 2013)) with the “--anysymbol” option;
• trimal (version 1.2rev59, (Capella-Gutiérrez et al., 2009)) with the “-gappyout” option;
• RAxML (version 8.1.15, (Stamatakis, 2014)) with parameters: “-m PROTCATLG -p 1989.”

The phylogeny in Figure S3A was built using the 400 PhyloPhlAn markers with the following parameters: “--diversity high --fast --min_num_markers 80” and the set of external tools with the same options used for the phylogeny in Figure 1A described above, except for the phylogeny reconstruction step. In this case the phylogeny has been inferred using IQ-TREE (version 1.6.6, (Nguyen et al., 2015)) with the following parameters: “-nt AUTO -m LG.”

The phylogenies in Figures 3C, S3B, S3C, S5, and S7B were built using their corresponding set of cores genes at 95% as identified by Roary (Page et al., 2015) and with the following parameters in PhyloPhlAn: “--diversity low --fast --min_num_markers <50% of the number of core genes identified>--min_num_entries <90% of the number of input genomes>.” The external tools used by PhyloPhlAn and their corresponding parameters were:

• blastn (version 2.6.0+, (Altschul et al., 1990)) with parameters: “-outfmt 6 -max_target_seqs 1000000”;
• mafft (version v7.310, (Katoh and Standley, 2013)) using the “L-INS-i” algorithm and with parameters: “--anysymbol --auto”;
• trimal (version 1.2rev59, (Capella-Gutiérrez et al., 2009)) with the “-gappyout” option;
• FastTree (version 2.1.9, (Price et al., 2010)) with “-mlacc 2 -slownni -spr 4 -fastest -mlnni 4 -no2nd -gtr -nt” options;
• RAxML (version 8.1.15, (Stamatakis, 2014)) with parameters: “-p 1989 -m GTRCAT -t <phylogenetic tree computed by FastTree>.”

The non-metric multidimensional scaling plots in Figures 4C and S6A were computed on pairwise genetic distances between core gene alignments produced by Roary using the nmds function in the ecodist R package (Goslee and Urban, 2007)

The phylogenetic trees in Figures 1A, 3C and S3A were generated using GraPhlAn (version 1.1.3, (Asnicar et al., 2015)) and the phylogenies in Figures 3A, S3B, S3C, S5, and S7B were generated using FigTree (version 1.4.3, http://tree.bio.ed.ac.uk/software/figtree/).

#### Quantification of the fraction of reads that can be mapped against SGBs

To assess the proportion of reads that could be mapped against the previously available set of genomes and the genomes we reconstructed here from metagenomes, we built four collections of sequences belonging to: a) the set of 12,563 genomes representing the kSGBs from the 80,990 reference genomes, by selecting one representative genome (the longest) for each kSGB; b) the residual set of 68,427 reference genomes for all the kSGBs; c) the set of 4,930 reconstructed genomes that are representatives for each SGB (Table S4); d) the residual set of 149,793 reconstructed genomes in all the SGBs. Additionally, we retrieved and indexed nine reference genomes for *Blastocystis* spp. (Beghini et al., 2017); 39 *Malassezia* spp. genomes from the NCBI-Assembly database (accessed in March 2018) and 18 assemblies from (Tett et al., 2017); and 13,924 plasmids and 10,529 viruses from RefSeq (release 90 (O’Leary et al., 2016)). To parallelize the downstream analysis and keep reasonably small the index files, 379 Bowtie2 (Langmead and Salzberg, 2012) databases were built. We then subsampled all the 9,428 samples used in this study to 1%, because of the very high computational requirement of the mapping (∼1,100 CPU hours for each sample would be required for the mapping of full metagenomes). The raw reads were filtered to remove short reads (length lower than 70 bp) and low-quality reads (mean sequencing quality < 20). We mapped each sample against the human genome using Bowtie2 (in end-to-end mode, hg19 index) to remove human DNA contamination and samples harboring more than 10% human reads were excluded. We excluded duplicated samples present in multiple studies, and samples that, after the quality-filtering, had no remaining reads. The reads from the remaining 8,908 samples were then mapped against the 379 Bowtie2 indexes in end-to-end mode. We applied the same procedure to the 389 additional cross-validation samples (384 publicly available, see above, and 5 sequenced gut metagenomes from Ethiopia). The resulting maping files were filtered to remove alignments with an alignment score (AS:i tag) lower than −20 to exclude spurious alignments that could influence the mappability assessment. For each sample, we computed the fraction of reads confidently mapping to each set of indexes and counted them according to the following criteria: **i)** reads aligning to at least one representative reference genome; **ii)** reads not aligning to i) and aligning to at least one other reference genome; **iii)** reads not aligning to i) and ii) and aligning against one of the 4,930 SGBs representatives; and **iv)** reads aligning only against one of the residual 149,793 reconstructed genomes. We followed the same incremental strategy to determine the fraction of residual reads mapping to micro-eukaryotes (*Blastocystis* spp., *Malassezia* spp.), plasmids and viruses. We reported in Figures 2A–2B and in Figure S4 the percentage of reads in each of these four categories (representative reference genomes, other reference genomes, representative SGBs and non-representative SGBs) with respect to the number of HQ non-human reads in each sample.

#### Pangenome, phylogenetic, and functional analysis of kSGB and uSGBs

We used Prokka (version 1.12, (Seemann, 2014), with default parameters) for annotating the reference and the reconstructed genomes of the 10 *Bacteroides* kSGBs. The annotated genomes were then processed with Roary (version 3.8, (Page et al., 2015) with “-e -z -g 1000000” params) for the pangenome analysis and to identify the set of core genes. The core genes (at 95% gene family clustering identity threshold) identified by Roary were then used as a database in PhyloPhlAn for phylogenetic analyses. Functional annotation was performed using EggNOG mapper (version 1.0.3, (Huerta-Cepas et al., 2017)) based on EggNOG orthology data (Huerta-Cepas et al., 2016), and the sequence searches were performed using HMM. For the functional profiles shown in Figures 3E and 5E, we used the Brite Hierarchy from KEGG to screen metabolic related pathways and KOs among all the KOs annotated by EggNOG. We employed the same EggNOG pipeline to functionally annotate all the 4,930 representative of the SGBs (Table S4). Figure S1A shows, based on the presence/absence of the EggNOG ortholog to which a KEGG KO is associated, an ordination plot relating each of the 4,930 SGBs from the functional point of view. All the 154,723 reconstructed genomes were functionally annotated by mapping them against Uniref90 and Uniref50 using diamond (version v0.9.9.110). The UniRef-based functional profiles are shown in the ordination plot in the Figures S1B–S1D. All functional profiles (EggNOG-based and UniRef-based) are available for download at the supporting website (see Data and software availability).

### Quantification and statistical analysis

Statistical significance was verified through Fisher's test, Mann-Whitney U-test, or Welch’s t-test as reported in the text. Multiple hypothesis testing correction was done using the Bonferroni or the false discovery rate (FDR) method as also reported in the manuscript. All other computational and statistical analyses were performed with the open source software tools referenced in the STAR Methods along with the described procedures.

### Data and software availability

All the recovered genomes, SGBs, and functional profiles (eggNOG- and UniProt-based) are available at http://segatalab.cibio.unitn.it/data/Pasolli_et_al.html and at http://opendata.lifebit.ai/table/?project=SGB. The raw sequencing data for the sequenced datasets are available in NCBI-SRA under the BioProject: PRJNA485056 (Madagascar cohort) and PRJNA504891 (Ethiopia cohort). The proposed representative genome of “*Candidatus* Cibiobacter qucibialis” has been deposited at DDBJ/ENA/GenBank under the accession SAUS00000000, assembled from NCBI-SRA accession ERS1343406. The metadata for all the samples considered are available in *curatedMetagenomicData* (Pasolli et al., 2017) at http://waldronlab.io/curatedMetagenomicData/, and all the other considered genomes and metagenomes are publicly available in NCBI. We also included in the resource the list of 644 genomes that recently became available in NCBI and the link to their uSGBs. Assembled contigs are available at http://segatalab.cibio.unitn.it/data/Pasolli_et_al.html, and software generated in this study is open source and available at https://bitbucket.org/CibioCM/cmseq/src/default/.

## Supplemental Information

Table S1. Description of the Datasets and Samples Used in This Study, Related to Figure 1 (Tab 1) List and description of the public datasets used in this study. We report relevant metadata such as body site, age categories considered, lifestyle, number of samples, and number of reconstructed genomes. (Tab 2) The complete list of public and additionally sequenced metagenomes used in this study. Number of reads and their median length, number of bases, and relevant metadata (body site, age, lifestyle, country) are reported. (Tab 3) Sequencing statistics and relevant metadata for the 112 stool metagenomes we sequenced from the MAHERY cohort in Madagascar. (Tab 4) List and description of the “validation” datasets used in this study. These datasets were not considered for SGBs reconstruction, but used to demonstrate the high increase in terms of raw-read mappability.

Table S2. Quality of the Single-Sample Assembled Genomes Compared against Multiple Alternative Genome Reconstruction Approaches, Related to Figure 7 (Tab 1) Comparison between the genomes sequenced from isolates and genomes reconstructed from the metagenomes sequenced from the same specimens or individuals from which the isolate was retrieved. The comparison is done for 8 different species (6 *Bifidobacterium* species, *Staphylococcus aureus*, *Pseudomonas aeruginosa*) from 3 cohorts. (Tab 2) Evaluation of the automatic genome reconstruction and binning pipeline adopted here with respect to binning aided by manual curation (using anvi’o). For 46 genomes reconstructed with anvi’o, our automatic binning procedure recovered a genome in all 46 cases and we report genome characteristics for both approaches such as genome size, N50, number of contigs, completeness, contamination, and alignment coverage computed with the reconstructed genome from anvi’o against the automatic binning (and vice versa). (Tab 3) Comparison between the set of single-sample assembled genomes and co-assembled genomes obtained from from the gut metagenomes of 25 adult women from the FerrettiP_2018 dataset (Ferretti et al., 2018). (Tab 4) Comparison of co-assembly against single-sample assembly and binning across 26 longitudinal and one cross-sectional sample subsets. We report the subject ID in the original study (Subj_Id), the number of samples in co-assembly (NSamples), the number of 1% core ANI CONCOCT genomes with more than 75% single copy core genes (nrGENs_C), the number of 1% core ANI single sample genomes (nrGENs_S), the number of genomes found by both methods (nrGENs_I), and the fractional increase in genome number from co-assembly (Frac).

Table S3. Assembly Statistics and Quality Control Measures for the 154,723 Reconstructed Genomes, Related to Figure 6

Table S4. Description of the Reconstructed SGBs, Related to Figure 1 (Tab 1) List and description of the reconstructed kSGBs and uSGBs, together with number of reconstructed and reference genomes belonging to each SGB, and estimated taxonomy with the distance from the closest reference genome. (Tab 2) Estimated abundances for each SGB, computed by extracting the coverage of each reconstructed genome from the mapping of the reads of each sample against its corresponding metagenomic assembly. (Tab 3) List of the representative genomes selected for each of the 4,930 SGBs.

Table S5. List of UniRef50 Gene Families that Are Statistically Significantly Different between Host Categories, Related to Figure 1 List of statistically significant UniRef50 annotated genes (Fisher's test, Bonferroni-corrected, p < 0.01) for age category, body site, and westernization categories.

Table S6. Protocols, SGBs, and Functional Profiles Associated with the Non-Westernized Cohorts, Related to Figure 5 (Tab1) Protocols applied in studies focusing on non-Westernized and Chinese cohorts, showing big differences in sample storage conditions, and sequencing approaches used. (Tab 2) SGBs associated with the Madagascar cohort. Bonferroni-corrected p values and number of reconstructed genomes belonging to each SGB are reported. (Tab 3) List of SGBs associated with non-Westernized metagenomes. Bonferroni-corrected p values and number of reconstructed genomes belonging to each SGB are reported. (Tab 4) Complete table of metabolism-related KEGG molecular functions of a set of representative genomes for 50 SGBs significantly associated with Westernized and non-Westernized populations (25 SGBs most associated with Westernized and 25 with non-Westernized were considered). To ensure an equal weight for all species in the comparison three randomly genomes were selected for each SGB (reduced version in Figure 5E).

Table S7. Table S7: “KEGG Molecular Functions for *Ca*. Cibiobacter qucibialis, Related to Figure 3 Complete table of metabolism-related KEGG molecular functions for *Ca.* Cibiobacter qucibialis (uSGB 15286, reduced version in Figure 3E).
